# Microbial embryonal colonization during pipefish male pregnancy

**DOI:** 10.1038/s41598-018-37026-3

**Published:** 2019-01-09

**Authors:** Anne Beemelmanns, Maude Poirier, Till Bayer, Sven Kuenzel, Olivia Roth

**Affiliations:** 1 GEOMAR Helmholtz-Centre for Ocean Research Kiel, Evolutionary Ecology of Marine Fishes, Düsternbrooker Weg 20, 24105 Kiel, Germany; 2 Max-Planck Institute for Evolutionary Biology, August-Thienemann-Str. 2, 24306 Plön, Germany

## Abstract

While originally acquired from the environment, a fraction of the microbiota is transferred from parents to offspring. The immune system shapes the microbial colonization, while commensal microbes may boost host immune defences. Parental transfer of microbes in viviparous animals remains ambiguous, as the two transfer routes (transovarial *vs*. pregnancy) are intermingled within the maternal body. Pipefishes and seahorses (syngnathids) are ideally suited to disentangle transovarial microbial transfer from a contribution during pregnancy due to their maternal egg production and their unique male pregnancy. We assessed the persistency and the changes in the microbial communities of the maternal and paternal reproductive tracts over proceeding male pregnancy by sequencing microbial 16S rRNA genes of swabs from maternal gonads and brood pouches of non-pregnant and pregnant fathers. Applying parental immunological activation with heat-killed bacteria, we evaluated the impact of parental immunological status on microbial development. Our data indicate that maternal gonads and paternal brood pouches harbor distinct microbial communities, which could affect embryonal development in a sex-specific manner. Upon activation of the immune system, a shift of the microbial community was observed. The activation of the immune system induced the expansion of microbiota richness during late pregnancy, which corresponds to the time point of larval mouth opening, when initial microbial colonization must take place.

## Introduction

Microbes can be parasites that have a detrimental impact on their hosts. However, a vast diversity of non-pathogenic microbes lives as commensals or mutualists on their hosts. Altogether they constitute the “holobiont”^1–4^. As specialized entities within organs^5^, microbiota shape almost every aspect of animal physiology (behaviour, reproduction, fitness) and may even play a role in hybridization and speciation^6–10^. For the successful host development, microbiota are indispensable, as they foster the production of polysaccharides and vitamins, boost the maturation of the host immune system, protect the host against invasions by pathogens, and maintain host tissue homeostasis^11–16^.

In contrast to maternal and paternal genes that are inherited in a Mendelian manner, the microbiome was originally acquired from the environment. Its transfer from parents to offspring revitalizes aspects of Lamarckism^4^. Siblings have a more similar microbiome than unrelated newborns, and a litter harbours offspring with more similar microbiota than offspring from different litters^17–20^. This implies that both the environment and the genetic background shape the early microbiome composition^21,22^. The maternal environmental experience may thus modulate the embryonic immune system in two ways: via the direct transfer of immunity^23^ and via the transfer of commensal bacteria^24^. This suggests a co-adaptation between parental investment, the host microbiota and the immune system.

Several features make the sex-role reversed pipefish *Syngnathus typhle* ideal for studying microbial transfer from parents to offspring. While pregnancy has evolved multiple times independently in most vertebrate groups^25,26^, syngnathids (pipefishes and seahorses) are the unique representative of male pregnancy evolution^27,28^. Mothers transfer their eggs into the paternal brood pouch, which is connected to a placenta-like system, where they are bred for 4–6 weeks^28,29^. We predict that in pipefish both vertical maternal transmission of specific commensal bacteria and additional paternal transmission during male pregnancy may shape initial translocation of embryonic microbiota and influence immunological maturation. This system permits the disentangling between transovarial microbial transfer (maternal) and microbial contribution during pregnancy (paternal), two traits that are in all other viviparous systems intermingled within the female.

In *S. typhle* both fathers and mothers contribute to the maturation of the offspring immune system^30–32^. Upon activation of parental immune defence using injections of heat-killed bacteria, offspring elevated their immune responses independent of whether only mothers, fathers or both were immunologically activated, which suggests biparental transfer of immunological parental experience^30^. While mothers rather influenced the adaptive immune system, fathers had a stronger impact on offspring innate immune responses^32^. We thus hypothesize that the parental immune system could also interact in a sex-specific way with the microbiome in the ovaries and in the brood pouch, which may result in a modulation of offspring immunological maturation. Such co-adaptation between microbiota and the vertebrate immune system not only enables the clearance of potentially virulent pathogens but also the persistence of specific mutualistic microbial communities^33^. The extent to which the immune system is able to control the microbiome still remains subject to current research and discussion^34–36^.

**Figure 1:**
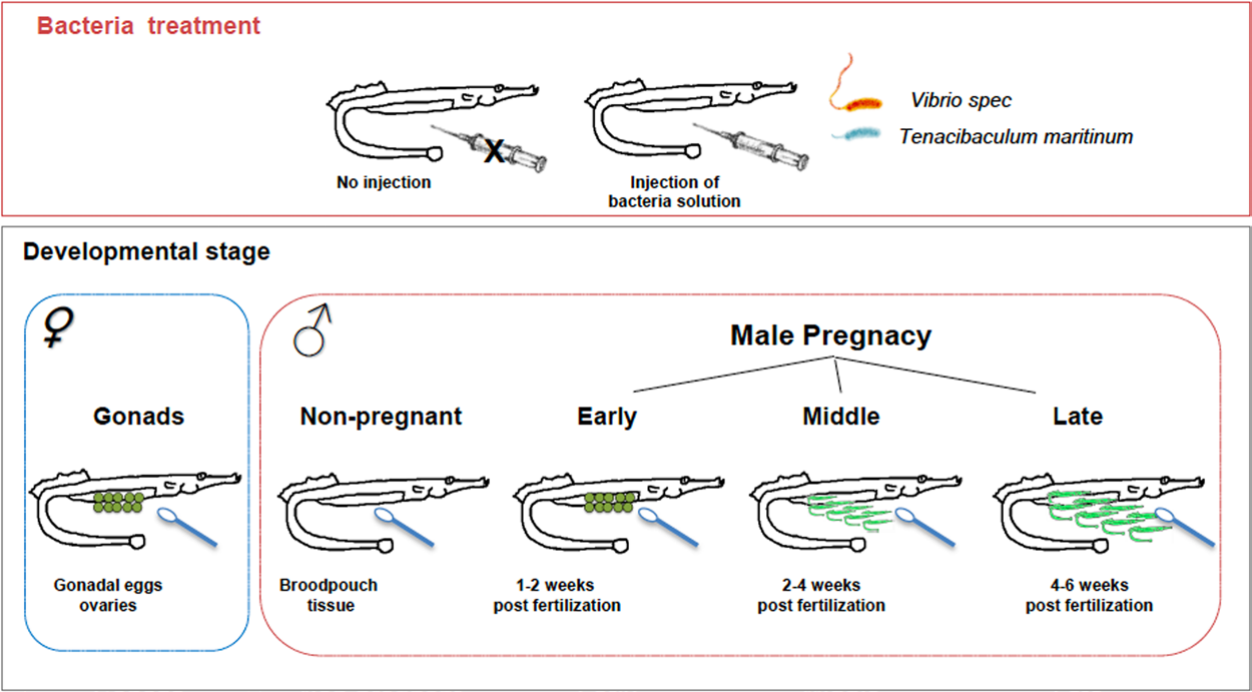
Sampling overview. Swab samples were taken from ovaries dissected out of pipefish (*Syngnathus typhle*) females (Gonads-GO); from the brood pouch tissue of non-pregnant pipefish males (Non-Pregnant-NP); on the surface of eggs and embryos inside the brood pouch of pregnant males in increasing developmental stages: early pregnancy (EP) circa (1–2 weeks); mid pregnant (MP) (2–4 weeks); late pregnant (LP) (4–6 weeks). In total 20 females, 20 males per pregnancy stage (3*20), and 40 non-pregnant males were used for the study (120 fish). For each group half of the individuals were intraperitoneally injected with heat-killed bacteria suspension (*Vibrio* spp. and *Tenacibaculum maritimum*) to trigger a parental immune transfer (injected with bacteria solution vs. non-injected). Microbiota samples were taken with sterile swabs by scratching the mucus from the surface of ovaries, brood pouch tissue and embryos inside the paternal brood pouch. Microbiota analysis was performed by Illumina Miseq Sequencing.

We aimed to describe differences in the microbiome (β-diversity by calculating Bray-Curtis matrix) and microbial diversity (α-diversity based on species richness (number of observed OTUs), estimated species richness (Chao Index) and species diversity (Shannon Index and inverse Simpson)) over the embryonal development in the pipefish *Syngnathus typhle*. We genotyped the microbial 16S rRNA gene and analysed its diversity on the surface of the developing eggs in the female ovaries, in the developed brood pouch of non-pregnant males, and on the surface of embryos in the brood pouch of pregnant males during early, mid and late pregnancy (Fig. 1). To determine the role of the parental immunological status in shaping microbial communities, parental immunity was either activated with heat-killed bacteria treatment or animals were left naive. The results from this study give insight into microbial communit changes from egg production to the embryo in different stages of pregnancy in a sex-role reversed fish. This also sheds light on the interaction between parental immunological status and the microbiota development in the female reproductive organ, for egg production and in the male brood pouch, for embryo development. This information will be useful for future experiments to disentangle maternal (egg production) and paternal (pregnancy) microbiota contribution towards the development of the offspring microbiome.

## Results

### Distinct microbiomes in developmental stages, depending on parental immunological status

**Figure 2:**
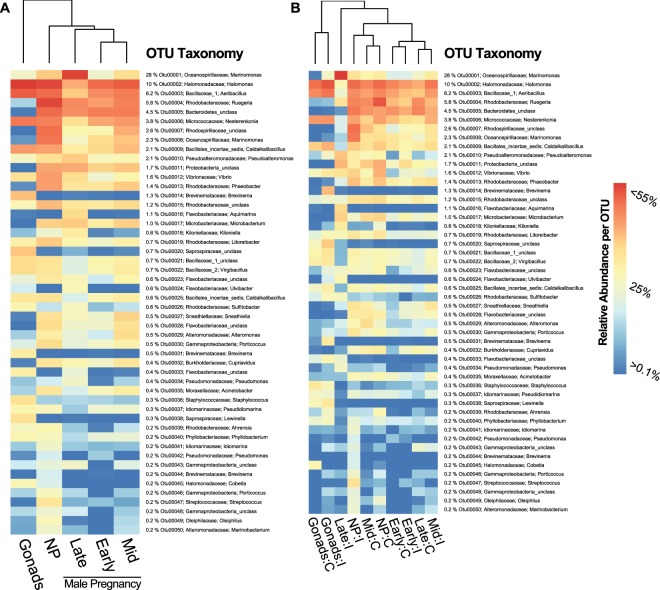
Clustered and hierarchical ordered heatmaps of the 50 most abundant OTUs (>90%) based on relative abundance for each individual OTU to identify connection between occurrence of most abundant species (OTUs) and surface of gonads and fertilized eggs/embryos of increasing male pregnancy inside the paternal brood pouch. Number of top 50 OTUs, their individual relative abundance in relation to all detected OTUs in [%] and classifications of phylum and species level are provided on the right side of the heatmap. Panel (**A**) Heatmap is clustered according to similarities between following treatment groups: gonadal eggs (Gonads), brood pouch tissue of non-pregnant males (NP), paternal brood pouch with increasing male pregnancy (Early, Mid, Late). Panel (**B**) Heatmap is clustered according to similarities of developmental stages x bacterial treatment interaction terms (Gonads:Control, Gonads:injected, Early:Control, Early:injected, Mid:Control, Mid:injected, and Late:Control, Late:injected, Non-Pregnant:Control, Non-Pregnant:injected).

In total, 994,755 16S rRNA sequences were retained from Illumina Miseq sequencing platform after merging and quality control. Across 77 samples (gonads (GO): 16; non-pregnant (NP): 25; early pregnancy (EP): 9; mid-pregnancy (MP): 15; late pregnancy (LP): 12, Supplemental Table S1), a total of 3090 OTUs (97% cut-off) were identified. Negative controls used in the PCRs showed no bands on the agarose gels and were thus not included for sequencing. The 50 most abundant OTUs of all samples (the first 50 most abundant OTUs contribute in total to 90% of the microbial communities) included members of Proteobacteria (Alpha-, Beta- and Gamma), Bacteroidetes (Flavobacteria, Sphingobacteria), Firmicutes (Bacilli), Actinobacteria and Spirochaetes (Supplemental Table S2). The distribution of the ten most abundant OTUs were Otu00001: Oceanospirillaceae, *Marinomonas* (28.2%), Otu00002: Halomonadaceae, *Halomonas* (10.3%), Otu00003: Bacillaceae_1, *Aeribacillus* (6.2%); Otu00004: Rhodobacteraceae, *Ruegeria* (5.8%), Otu00005: Bacteroidetes_unclassified (4.5%); Otu00006: Micrococcaceae, *Nesterenkonia* (3.8%), Otu00007: Rhodospirillaceae_unclassified (2.6%); Otu00008: Oceanospirillaceae, *Marinomonas* (2.3%), Otu00009: Bacillales_incertae_sedis, *Caldalkalibacillus* (2.1%); Otu00010: Pseudoalteromonadaceae, *Pseudoalteromonas* (2.1%) (Fig. 2, Supplemental Table S2). Based on the most abundant 50 OTUs, the microbiota clustered according to the developmental stage of the fishes. The gonad microbiota differed from the paternal brood pouch microbiota (Fig. 2A). Brood pouch microbiota of non-pregnant males differed from both the gonad and pregnancy associated microbiota, while it was more similar to non-pregnant brood pouch microbiota (Fig. 2A). Microbiota in the brood pouch of late-pregnant males was distinct from earlier pregnancy stages (Fig. 2A). In addition, brood pouch microbiota during late stage pregnancy showed a distinct clustering of microbiota, when their parents had experienced an injection with heat-killed bacteria (Fig. 2B). The most abundant Otu00001: Oceanospirillaceae, *Marinomonas* with 28% was overrepresented in late pregnancy brood pouch microbiota (Fig. 2A), in particular if fathers were exposed to prior immune challenges (Fig. 2B). In contrast, the second and third most abundant OTUs Halomonadaceae, *Halomonas* with 10.3% and Bacillaceae_1, *Aeribacillus* with 6.2% were predominantly prevalent in the gonads of injected females (GO_I) (Fig. 2B). The microbiota in the brood pouch of non-pregnant, early, mid and late pregnant males were, independent of the paternal immunological activation, more uniform than the microbiota in the female gonads (Fig. 2). This could imply that eggs transferred from mothers into the paternal brood pouch came in contact with a novel microbial community, which is during late pregnancy subject to drastic changes.

### Bacterial α -diversity within and between developmental stages and the impact of parental immunological activation

For the assessment of bacterial community shifts within and between developmental stages on the surface of pipefish eggs/embryos during male pregnancy in combination with parental immunological activation, α-diversity indices based on species richness (number of observed OTUs), estimated species richness (Chao Index) and species diversity (Shannon Index and inverse Simpson) were analysed in linear mixed effect models^37^.

**Figure 3:**
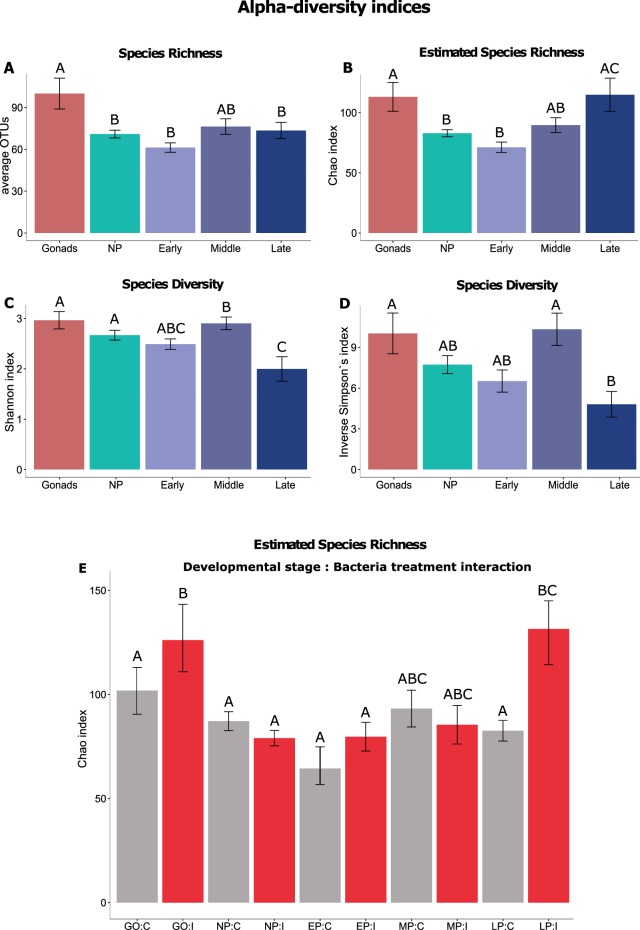
α-diversity comparison between different developmental stages and parental bacteria treatments. Illustrated are (**A**) bacterial species richness (Number of average 97%-OTUs), (**B**) estimated species richness (Chao) as well as (**C**) alpha diversity estimates inverse Simpson’s and (**D**) Shannon index of gonads and brood pouch microbiota with no or increasing pregnancy stage. (**E**) α-diversity Chao index demonstrating interaction effects. Illustrated are interactions between developmental stage and parental bacteria treatment (Gonads:Control, Gonads:injected, Early:Control, Early:injected, Mid:Control, Mid:injected, and Late:Control, Late:injected, Non-Pregnant:Control, Non-Pregnant:injected). Measurements of indices (average OTUs, Chao, Inverse Simpson, Shannon) were analysed using a linear mixed effect model followed by lsmeans post hoc test (Table 1, Supplemental Table 1). Significant differences between groups analyzed by pairewise comparisons (*p* < 0.05) are indicated with capital Letters (groups with a distinct letter (A,B,C) are significantly different from each other).

Species richness (numbers of OTUs) was significantly higher on gonadal eggs (GO) compared to brood pouch tissue of non-pregnant males (NP) and on pipefish embryos of early (EP) and late pregnant (LP) males (LMER*-#OTU*: F_4,67_ = 3.84*, p* = 0.007**; *TukeyHSD:* GO > NP; GO > EP; GO > LP, Table 1, Fig. 3A). Estimated species richness index (Chao-index) significantly differed among developmental stages (LMER*-Chao-dev. stage*: F_4,67_ = 3.93*, p* = 0.006**, Table 1). Both gonads and late pregnancy pouch samples revealed a high estimated species richness index (Fig. 3B), whereas pouch tissue of early pregnant and brood pouch tissue of non-pregnant males were in a graduate decline (*TukeyHSD-Chao-developmental stage:* GO > NP; GO > EP; EP < LP, Table 1, Fig. 3B). We detected an interaction between developmental stage and parental bacteria treatment (LMER*-Chao-dev. stage x bact. treat:* F_4,67_ = 2.53, *p* = 0.04*, Table 1). Gonad samples of injected females (I) and pouch tissue of injected pipefish males in late pregnancy stage both revealed a significantly higher estimated species richness in comparison to control animals without bacteria injection (C) (*TukeyHSD-Chao-dev. stage x bact. treat:* GO:C < GO:I; LP:C < LP:I, Table 1, Fig. 3E). In addition, an influence of developmental stage was detected on species diversity indices (LMER*-InSimpson*: F_4,67_ = 4.23*, p* = 0.004**; LMER*-Shannon*: F_4,67_ = 5.1*, p* = 0.001**, Table 1, Fig. 3C,D). Bacteria diversity (*Shannon index*) in pouch tissue of late pregnant males was lower than in pouch tissue of non- or mid pregnant males and in the gonads (TukeyHSD-*Shannon*: GO > LP; MP > LP; NP > LP, Table 1, Fig. 3C).

**Table 1:** Linear mixed effect model of α-diversity indices.

LMER	Term	NumDF	DenDF	F.value	P(>F)	Lsmeans Tukeys HSD post hoc test
OTUs	Bac.treat	1	67.00	0.83	0.366	
	Dev.stage	4	67.00	3.84	0.007**	**GO** > **NP; GO** > **EP; GO** > **LP**
	Bac.treat × Dev.stage	4	67.00	0.91	0.463	
Chao	Bac.treat	1	67.00	3.77	0.050	
	Dev.stage	4	67.00	3.93	0.006**	**GO** > **NP; GO** > **EP; EP** < **LP**
	Bac.treat × Dev.stage	4	67.00	2.53	0.04*	**GO:C** < **GO:I; LP:C** < **LP:I**; NP:C & NP:I < LP:I; EP:C & EP:I < LP:I
Inverse simpson	Bac.treat	1	4.24	0.03	0.870	
	Dev.stage	4	63.47	4.23	0.004**	**GO > LP; MP > LP**
	Bac.treat × Dev.stage	4	63.47	0.89	0.477	
Shannon’s	Bac.treat	1	4.5	0.2	0.671	
	Dev.stage	4	63.9	5.1	0.001**	**GO** > **LP; MP** > **LP;** NP > LP
	Bac.treat × Dev.stage	4	63.9	1.0	0.404	

LMER was performed on α-diversity indices (average OTUs, Chao, Inverse Simpson, Shannon) including the fixed factors parental bacteria treatment (Bac.treat), developmental stage (Dev. stage), their interaction effect and the random term (tank). Significant LMERs (P < 0.05, indicated in bold letters) were followed by lsmeans (glht function of multcomp package) as post hoc test that includes tank structure. Levels of parental bacteria treatment were: treatment control (C) and injected with heat-killed bacteria (I); Levels of developmental stages were: Gonads (GO), Non-pregnant (NP), early pregnancy stage (EP), mid (MP), and late pregnancy stage (LP) their interaction effect is indicated with a “:” symbol.

### Bacterial β-diversity within and between developmental stages and the influence of parental immunological activation

To examine differences in microbiota, we evaluated β-diversity with a Bray-Curtis matrix based on 1419 OTUs obtained by tools implemented in MOTHUR (subsamples > 6000). Bray-Curtis matrix was established on the ratio of shared and unique species relative to the total number of species abundance. We measured variance explained by ‘developmental stage’ and parental ‘bacterial treatment’ by applying a permutational multivariate analysis of variance (perMANOVA) in which ‘tank’ was included as random term by stratifying permutations 10000 times. The bacterial community structure was significantly different between specific developmental stages of gonads and brood pouches (perMANOVA-*Bray-Curtis*: stage: F_4,67_ = 6.26, *p* < 0.001***, R^2^ = 0.24, Table 2). Overall, developmental stage explained 15–24% of total variance assigned to community structure and composition. In contrast, the parental bacteria treatment as single factor did not significantly influence microbial community composition (perMANOVA-*Bray-Curtis*: bac. treat: F_4,67_ = 2.19, *p* = 0.207, R^2^ = 0.02, Table 2). Yet, the significant interaction between developmental stage and parental immunological activation (‘bacteria treatment’) reveals that the parental immune system influenced the gonad and pouch-specific microbiota (PERMANOVA-*Bray-Curtis:* dev. stage x bac. treat: F_4,67_ = 2.03, *p* = 0.001**, R^2^ = 0.08, Table 2).

**Table 2:** Permutation multivariate analysis of distance matrix (Bray-Curtis).

	Model	Dev. stage			Bact. treat			Dev. stage x Bac. treat		
Matrix	Residuals	F.Model	R2	P(>F)	F.Model	R2	P(>F)	F.Model	R2	P(>F)
BrayCurtis	0.655	6.26	0.24	<0.001***	2.19	0.02	0.207	2.03	0.08	0.001**
Residual Degrees of Freedom	DF = 67	DF = 4			DF = 1			DF = 4		
Total Degrees of Freedom	DF = 76									

Multivariate PERMANOVA analysis to assess the effect and interaction of the two fixed factors developmental stage (Dev. stage) x parental bacteria treatment (Bac. treat) while implementing tank as strata term with p-values obtained by 10000 permutations. Significant p-values are marked in bold letters (significance code: <0.001***, 0.001**, 0.01*). Each analysis was based on OTU distance matrices obtained by tools implemented in MOTHUR (1419 OTUs, subsamples > 6000).

**Figure 4:**
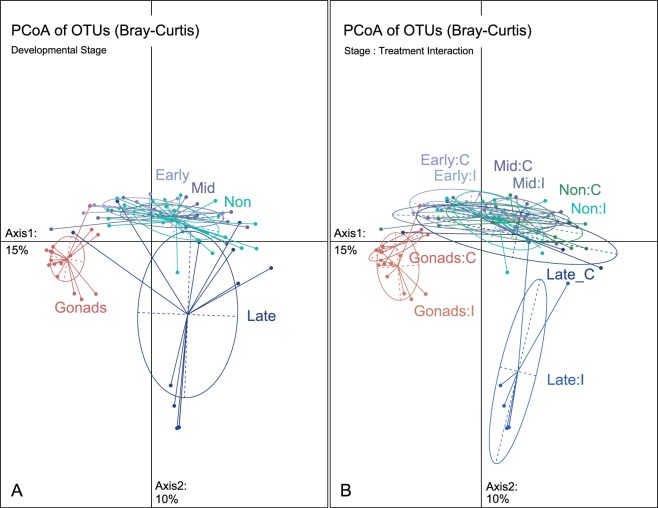
Principal Coordinate analysis (PCoA) of bacterial β-diversity based on OTU abundance (Bray-Curtis distance matrix) demonstrating bacterial community structure. Panel (**A**) PCoA of different developmental stages: gonadal eggs (Gonads), brood pouch tissue of non-pregnant males (Non), paternal brood pouch with increasing male pregnancy (Early, Mid, Late) (**B**) PCoA according to interaction between developmental stages x bacterial treatment interaction (Gonads:Control, Gonads:injected, Early:Control, Early:injected, Mid:Control, Mid:injected, and Late:Control, Late:injected, Non-Pregnant:Control, Non-Pregnant:injected). Ellipses denote the standard deviation around the mean of the respective group. Variance explained by PCoA Axis1: 15%; Axis2: 10%).

An ordination of principal coordinate analysis (PCoA) was applied on OTU abundance (Bray Curtis) measurements for the visualization of each developmental stage (Fig. 4). Subsequently, a linear mixed-effect model (type III sum of squares) was calculated for the first two extracted principle coordinates and significant axes were followed by *Tukey HSD* post-hoc tests to attain pairewise comparisons between specific treatment groups. In the two-dimensional PCoA depiction (Fig. 4A), the gonads revealed significantly different bacterial communities, they are the most distinct cluster that is set apart along the first principle component (explains 15% of variance) and cluster opposite from all other groups (LMER-*Bray-Curtis* Axis1: F_4,67_ = 17.1, *p* < 0.001***; *TukeyHSD:* GO vs NP, EP, MP & LP, Table 3, Fig. 4A,B). Likewise, late pregnancy brood pouch microbiota significantly sets apart along the second principle component (explains 10% of variance), yet it revealed an enormous variance by forming a large ellipse around its centroid rather than a homogeneous cluster (LMER-*Bray-Curtis* Axis2: F_4,67_ = 17.1, *p* < 0.001***; *TukeyHSD:* LP vs GO, NP, EP & MP, Table 3, Fig. 4). In contrast, the microbiota of brood pouch tissue of non-pregnant males (NP), early (EP) and mid (MP) pregnant males revealed overlapping centers of gravity and were not significantly different from each other (Table 3, Fig. 4).

**Table 3:** Results from linear mixed-effect model on the scores of three extracted principle coordinates based on Bray Curtis distance matrix.

Bray-Curtis	Axis1 (variance: 15%)				TukeyHSD post-hoc
		DenDF	F.value	P(>F)	
	Dev. stage	67.0	17.1	<0.001***	GO vs NP, EP,MP, LP
	Bact. treat	67.0	0.1	0.736	
	Dev. stage x Bact. treat	67.0	0.9	0.445	
	**Axis2 (variance 10%)**				
	Dev. stage	67.0	21.4	<0.001***	GO vs EP,MP, LP, NP and LP vs EP,MP,NP
	Bact. treat	67.0	17.4	<0.001***	C vs I
	Dev. stage x Bact. treat	67.0	12.6	<0.001***	EP:C vs. GO:I; EP:C vs. LP:I; GO:C vs. LP:I; LP:C vs. LP:I; MP:C vs. GO:I; MP:C vs. LP:I; NP:C vs. GO:I; **NP:C vs. LP:I**; EP:I vs. LP:I; GO:I vs. LP:I; GO:I vs. MP:I; GO:I vs. NP:I; LP:I vs. MP:I; LP:I vs. NP:I

Data reducing technique based on Bray Curtis (community structure) distance matrix to assess the effect and interaction of the two fixed factors developmental stage (Dev. stage) × parental bacteria treatment (Bact.treat) including tank as random factor. LMER was performed with type III sum of squares and Satterthwaite approximation for degrees of freedom. Significant values are marked in bold letters (significance code: <0.0001***, 0.001**, 0.01*). TukeyHSD post-hoc t-test of the linear mixed effect model was performed with ‘lsmeans’ to investigate pairwise comparison of corresponding levels of the fixed factors ‘Developmental stage’ (Early, Mid and Late Pregnancy stage, Non-pregnant (NP), Gonads of Females) and ‘Bacterial treatment’ (no injection (C), injection (I)). Each analysis distance matrices are based on all OTUs (1419, subsamples > 6000).

We found a developmental stage x parental bacteria treatment interaction effect on the second axis of the PCoA that displays the OTU abundance (LMER-*Bray-Curtis* Axis2: F_4,67_ = 12.6, *p* < 0.001*** Table 3, Fig. 4). The PCoAs indicate a different ß-diversity structure in the brood pouch during late pregnancy when males had received an immunological activation (Fig. 4). Only late pregnancy brood pouch samples of animals exposed to parental bacteria treatment (Late pregnancy: Injected ‘LP:I’) showed a significantly different microbiota in comparison to the respective control group (Late pregnancy: Control ‘LP:C’) (Fig. 4). To this end, the microbiota on the embryo in late pregnant immunologically activated males (LP:I) formed the most dissimilar cluster that is set apart along the second principle component (LMER-*Bray-Curtis* Axis2: *TukeyHSD:* LP:I vs LP:C and all other groups, Table 3, Fig. 4).

### Microbiota community compositions according to developmental stage and parental immunological activation

**Figure 5:**
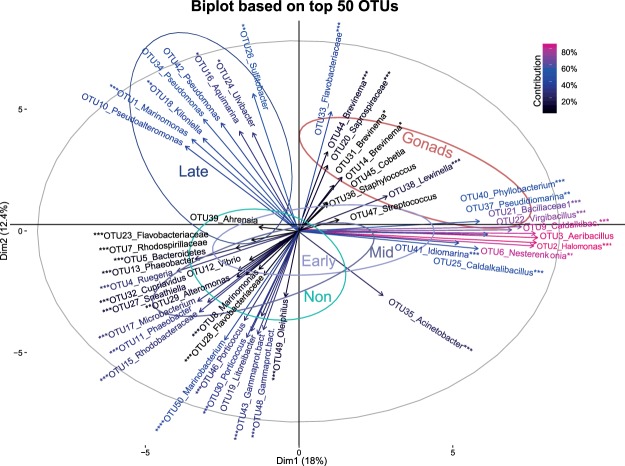
Biplot of the 50 most abundant OTUs (90%) to identify the correlation between occurrence of most abundant bacteria (OTUs) on the surface of gonads and in the brood pouch of increasing male pregnancy (Early, Mid, Late) as well as in the brood pouch of non-pregnant males (NP). Illustrated is a principle component analysis (PCA) with superimposed factormap. Ellipses demonstrate 95% confidence interval of centre of gravity of each developmental stage (Gonads; NP, Early, Mid, Late). Superimposed Factor map demonstrate the contribution of variance retained by each bacteria species (OTUs-genus level). The response variables (OTUs-genus level) are symbolized by arrows whereby the length of the arrow is directional proportional to the contribution of variance of each variable to the total variability. The colour gradient in the left corner highlights the importance of the bacteria species in explaining the variations (importance%) retained by the principle components calculated according to^88^. Asterix symbol behind OTU number *^,^**^,^*** demonstrate significant Indicator species according to indicator species analysis (Table 4).

Indicator species analyses^38^ and biplot were performed to identify and visualize associations between most abundant microbial species (OTUs) and particular pipefish developmental stages. The biplot consist of a principle component analysis that visualizes a differential clustering pattern with a superimposed factormap demonstrating the contribution of variance (importance %) retained by each bacteria species (Fig. 5). Results of indicator species analysis conducted using all OUT’s can be found as Supplemental Table S3, however, we limited the interpretation of data on the most abundant 50 OTUs. Among those we identified five significant OTUs as indicators for gonadal eggs and three for the late pregnancy stage (Table 4). On gonadal egg surface *Brevinema* bacteria were overrepresented (*Brevinema* Otu00044, Otu00031, Otu00014), but also *Lewinella* (Otu00038) and *unclassified_Saprospiraceae* (Otu00020) were highly abundant indicators species (Table 4, Fig. 5). In contrast, *Kiloniella* (Otu00018), *Aquimarina* (Otu00016), and *Ulvibacter* (Otu00024) were highly abundant on the surface of late pregnancy stage embryos (Table 4, Fig. 5). *Marinomonas* (Otu00001) is an indicator species on the surface of embryos during early and late pregnancy stage (Table 4, Fig. 5), whereas *Sulfitobacter* (Otu00026) and *Flavobacteriaceae_unclassified* (Otu00033) were abundant on the surface of gonads and embryos during late pregnancy stage (Table 4, Fig. 5).

**Table 4:** Indicator species analysis.

Dev. stage/ Bact. treat	OTUs	OTU Taxonomy					A	B	Stats	P value	
Gonads (GO)	**Otu00020**	Bacteroidetes	Sphingobacteria	Sphingobacteriales	Saprospiraceae	unclassified	0.98	0.94	0.96	0.001	***
	**Otu00038**	Bacteroidetes	Sphingobacteria	Sphingobacteriales	Saprospiraceae	***Lewinella***	1.00	0.56	0.75	0.001	***
	**Otu00044**	Spirochaetes	Spirochaetes	Spirochaetales	Brevinemataceae	***Brevinema***	0.89	0.44	0.62	0.001	***
	**Otu00031**	Spirochaetes	Spirochaetes	Spirochaetales	Brevinemataceae	***Brevinema***	1.00	0.31	0.56	0.014	*
	**Otu00014**	Spirochaetes	Spirochaetes	Spirochaetales	Brevinemataceae	***Brevinema***	1.00	0.25	0.50	0.044	*
Late (LP)	**Otu00018**	Proteobacteria	Alphaproteobacteria	Kiloniellales	Kiloniellaceae	***Kiloniella***	0.83	0.58	0.70	0.003	**
	**Otu00016**	Bacteroidetes	Flavobacteria	Flavobacteriales	Flavobacteriaceae	***Aquimarina***	1.00	0.25	0.50	0.021	*
	**Otu00024**	Bacteroidetes	Flavobacteria	Flavobacteriales	Flavobacteriaceae	***Ulvibacter***	0.87	0.25	0.47	0.031	*
Early + Late (EP + LP)	**Otu00001**	Proteobacteria	Gammaproteobacteria	Oceanospirillales	Oceanospirillaceae	***Marinomonas***	0.89	0.90	0.90	0.003	***
Gonads + Late (GO + LP)	**Otu00026**	Proteobacteria	Alphaproteobacteria	Rhodobacterales	Rhodobacteraceae	***Sulfitobacter***	0.85	0.79	0.82	0.002	**
	**Otu00033**	Bacteroidetes	Flavobacteria	Flavobacteriales	Flavobacteriaceae	unclassified	1.00	0.61	0.78	0.001	***
NP + Early + Mid (NP + EP + MP)	**Otu00007**	Proteobacteria	Alphaproteobacteria	Rhodospirillales	Rhodospirillaceae	unclassified	0.92	0.94	0.93	0.001	***
NP + Early + Gonads + Mid (NP + EP + GO + MP)	**Otu00037**	Proteobacteria	Gammaproteobacteria	Alteromonadales	Idiomarinaceae	***Pseudidiomarina***	0.97	1.00	0.98	0.001	***
	**Otu00025**	Firmicutes	Bacilli	Bacillales	Bacillales_incertae_sedis	***Caldalkalibacillus***	0.95	1.00	0.98	0.001	***
	**Otu00021**	Firmicutes	Bacilli	Bacillales	Bacillaceae_1	unclassified	0.95	1.00	0.97	0.001	***
	**Otu00022**	Firmicutes	Bacilli	Bacillales	Bacillaceae_2	***Virgibacillus***	0.95	1.00	0.97	0.001	***
	**Otu00006**	Actinobacteria	Actinobacteria	Actinomycetales	Micrococcaceae	***Nesterenkonia***	0.93	1.00	0.97	0.001	***
	**Otu00009**	Firmicutes	Bacilli	Bacillales	Bacillales_incertae_sedis	***Caldalkalibacillus***	0.92	1.00	0.96	0.001	***
	**Otu00041**	Proteobacteria	Gammaproteobacteria	Alteromonadales	Idiomarinaceae	***Idiomarina***	0.95	0.97	0.96	0.001	***
	**Otu00040**	Proteobacteria	Alphaproteobacteria	Rhizobiales	Phyllobacteriaceae	***Phyllobacterium***	0.94	0.97	0.95	0.001	***
NP + Early + Mid + Late (NP + EP + MP + LP)	**Otu00035**	Proteobacteria	Gammaproteobacteria	Pseudomonadales	Moraxellaceae	***Acinetobacter***	0.98	0.95	0.96	0.001	***
	**Otu00004**	Proteobacteria	Alphaproteobacteria	Rhodobacterales	Rhodobacteraceae	***Ruegeria***	1.00	0.89	0.94	0.001	***
	**Otu00008**	Proteobacteria	Gammaproteobacteria	Oceanospirillales	Oceanospirillaceae	***Marinomonas***	1.00	0.87	0.93	0.001	***
	**Otu00030**	Proteobacteria	Gammaproteobacteria	Gammaproteobacteria	Gammaproteobacteria	***Porticoccus***	0.94	0.90	0.92	0.001	***
	**Otu00013**	Proteobacteria	Alphaproteobacteria	Rhodobacterales	Rhodobacteraceae	***Phaeobacter***	1.00	0.84	0.91	0.001	***
	**Otu00028**	Bacteroidetes	Flavobacteria	Flavobacteriales	Flavobacteriaceae	unclassified	1.00	0.82	0.91	0.001	***
	**Otu00029**	Proteobacteria	Gammaproteobacteria	Alteromonadales	Alteromonadaceae	***Alteromonas***	0.98	0.82	0.90	0.002	**
	**Otu00017**	Actinobacteria	Actinobacteria	Actinomycetales	Microbacteriaceae	***Microbacterium***	1.00	0.79	0.89	0.001	***
	**Otu00027**	Proteobacteria	Alphaproteobacteria	Sneathiellales	Sneathiellaceae	***Sneathiella***	1.00	0.77	0.88	0.001	***
	**Otu00046**	Proteobacteria	Gammaproteobacteria	Gammaproteobacteria	Gammaproteobacteria	***Porticoccus***	1.00	0.75	0.87	0.001	***
	**Otu00005**	Bacteroidetes	Bacteroidetes_unclassified	unclassified	unclassified	unclassified	1.00	0.75	0.87	0.001	***
	**Otu00043**	Proteobacteria	Gammaproteobacteria	unclassified	unclassified	unclassified	1.00	0.72	0.85	0.001	***
	**Otu00048**	Proteobacteria	Gammaproteobacteria	unclassified	unclassified	unclassified	0.99	0.72	0.85	0.001	***
	**Otu00049**	Proteobacteria	Gammaproteobacteria	Oceanospirillales	Oleiphilaceae	***Oleiphilus***	1.00	0.70	0.84	0.001	***
	**Otu00050**	Proteobacteria	Gammaproteobacteria	Alteromonadales	Alteromonadaceae	***Marinobacterium***	1.00	0.69	0.83	0.001	***
	**Otu00032**	Proteobacteria	Betaproteobacteria	Burkholderiales	Burkholderiaceae	***Cupriavidus***	1.00	0.62	0.79	0.001	***
	**Otu00011**	Proteobacteria	unclassified	unclassified	unclassified	unclassified	1.00	0.43	0.65	0.017	*
Gonads_Injected	**Otu00020**	Bacteroidetes	Sphingobacteria	Sphingobacteriales	Saprospiraceae	***Saprospiracea***	0.98	0.94	0.96	0.001	***
	**Otu00033**	Bacteroidetes	Flavobacteria	Flavobacteriales	Flavobacteriaceae	***Flavobacteriace***	1.00	0.74	0.86	0.001	***
	**Otu00026**	Proteobacteria	Alphaproteobacteria	Rhodobacterales	Rhodobacteraceae	***Sulfitobacte***	0.81	0.87	0.84	0.004	**
	**Otu00038**	Bacteroidetes	Sphingobacteria	Sphingobacteriales	Saprospiraceae	***Lewinella***	1.00	0.56	0.75	0.003	**
	**Otu00044**	Spirochaetes	Spirochaetes	Spirochaetales	Brevinemataceae	***Brevinema***	1.00	0.43	0.66	0.01	**
	**Otu00031**	Spirochaetes	Spirochaetes	Spirochaetales	Brevinemataceae	***Brevinema***	0.98	0.40	0.63	0.045	*
Late_Injected	**Otu00018**	Proteobacteria	Alphaproteobacteria	Kiloniellales	Kiloniellaceae	***Kiloniella***	0.80	0.86	0.83	0.002	**
	**Otu00034**	Proteobacteria	Gammaproteobacteria	Pseudomonadales	Pseudomonadaceae	***Pseudomonas***	0.67	1.00	0.82	0.01	**
	**Otu00042**	Proteobacteria	Gammaproteobacteria	Pseudomonadales	Pseudomonadaceae	***Pseudomonas***	0.90	0.71	0.80	0.007	**
	**Otu00016**	Bacteroidetes	Flavobacteria	Flavobacteriales	Flavobacteriaceae	***Aquimarina***	1.00	0.43	0.65	0.021	*
	**Otu00024**	Bacteroidetes	Flavobacteria	Flavobacteriales	Flavobacteriaceae	***Ulvibacter***	0.88	0.43	0.61	0.012	*

List of 97% OTUs associated to each combination (Indicator Species) to identify associations between species (OTUs) and combination of developmental stage and parental bacteria treatment: gonads (GO), paternal brood pouch with increasing male pregnancy (EP, MP, LP) as well as brood pouch of non-pregnant males (NP). Further Indicator species are listed that occurred on gonads and late pregnancy stage embryos of parents, which were injected with heat-killed bacteria solution (GO:Injected and LP:injected). An indicator species analysis was performed with the package ‘indicspecies’ implemented in R based on 1000 permutations^89^ on Top 50 OTUs abundance. Listed are the OTUs, the OTU Taxonomy, the indicator value (IndVal) index with is the product of A (specificity) and B (sensitivity) probabilities and significance values (p-values). Only OTUS with an IndVal value higher than > 0.5 (A and B) were considered to exclude bacterial taxa, which were only present in one or few developmental stage tissue samples.

Eight bacteria OTUs including *Nesterenkonia* (Otu00006), *Caldalkalibacillus* (Otu00009), *Bacillaceae_1_unclassified* (Otu00021), *Virgibacillus* (Otu00022), *Caldalkalibacillus* (Otu00025), *Pseudidiomarina* (Otu00037), *Phyllobacterium* (Otu00040), *Idiomarina* (Otu00041) were overrepresented on the gonads but also in the brood pouch of non-pregnant males and on the embryos during early and mid-pregnancy (EP and MP) in contrast to late pregnancy. In the biplot these OTUs describe the highest percentage of contribution (between 60–80%) in comparison to all other species.

We detected 19 specific OTUs as indicators of the brood pouch microbiota, i.e., the tissue of non-pregnant males and surface of eggs and embryos inside the paternal brood pouch during early, mid and late pregnancy (Table 4, Fig. 5). They belong to Proteobacteria such as *Rhodospirillaceae_unclassified (Otu00007)*, *Acinetobacter* (Otu00035), *Ruegeria* (Otu00004), *Marinomonas* (Otu00008), *Porticoccus* (Otu00030), and *Phaeobacter* (Otu00013) (see Table 4 for further species, Fig. 5). These might shape the microbiota inside the paternal brood pouch that incorporates the embryos during male pregnancy.

We performed a species indicator analysis for developmental stage in combination with parental bacteria treatment (interaction effect) and identified the following indicator species for gonads of females that received an immunological activation: *Brevinema* (Otu00044, Otu00031), *Lewinella* (Otu00038) and unclassified_*Saprospiraceae* (Otu00020) and unclassified_*Flavobacteriace* (Otu00033) and *Sulfitobacter* (Otu00026) (Table 4). The latter two species only occurred in combination with parental bacteria treatment. Also for late pregnancy stage of bacteria treated males, we found overlapping indicator species *Kiloniella* (Otu00018), *Aquimarina* (Otu00016), and *Ulvibacter* (Otu00024) but as a new species *Pseudomonas* (Otu00034, Otu00042) (Table 4).

## Discussion

In order to understand how microbes colonize reproductively important tissues, persist, change and may outcompete each other to finally shape offspring development and life history, we applied a 16S rRNA gene sequencing approach to identify and compare routes of initial microbial colonisation of embryos. We characterized the parental microbial community over the development of the egg in the gonads and embryo in the brood pouch of the pipefish *Syngnathus typhle* and assessed the influence of the immune system on microbial colonization during pregnancy by parental bacteria injections. The unique male pregnancy permits to assess maternal contribution through the eggs and paternal contribution during embryonic development separately. This sheds light on the interaction of microbial colonization and the parental immune system.

The most abundant bacteria in the maternal gonads and in the paternal brood pouch of *S. typhle* belong to *Marinomonas* (28.0%), *Halomonas* (10.3%), *Aeribacillus* (6.3%), *Ruegeria* (5.8%), *Bacteroidetes* (4.5%) and *Nesterenkonia* (3.8%). They are all known members of fish microbiota^39–43^. *Marinomonas* are important initial colonizers^41,44,45^, *Halomonas* are gastroinstestinal bacteria^43^ and *Ruegeria* are prevalent on cod larvae^39^.

During mammalian pregnancy the vaginal bacterial community shifts naturally in its structure with respect to diversity and richness^46,47^. The major shift in microbiota during late pregnancy in the pipefish *S. typhle* suggests the parallel evolution of a similar pattern in male pregnancy. The human vaginal microbiome of pregnant women was suggested to be distinct from non-pregnant women in terms of a decreased species diversity and due to an absence of occasionally present unique taxa^47^. The development of the pipefish brood pouch microbiota community goes in line with this observation: in the brood pouch of the pipefish *S. typhle* bacterial α *-*diversity decreased in late pregnancy, while the number of bacteria OTU identified (species richness) was higher. This enhanced variation in the composition of the microbiota suggests a major restructuring that probably facilitates the initial colonisation of the embryos. The passage of surface bacteria into the gut and early digestive tract has been shown to start in oviparous fish when larvae begin to ingest liquid medium^48,49^. Freshly hatched eggs in the brood pouch of the male pipefish (early pregnancy and mid pregnancy) are supplied with essential ions, oxygen and nutrition proteins over a placenta-like structure from the father in addition to the essential supply by the maternal yolk sac^50–53^. The embryonal development takes place in a paternally shaped environment. To this end, the embryos are supposedly soaked in a cocktail of paternal bacteria. This makes it tempting to speculate that once yolk sac supply is depleted, which usually corresponds to the opening of the larval mouth (i.e., between mid and late pregnancy in pipefish), the paternal microbial community are dominating the gut of pipefish larvae.

The bacterial community in the paternal brood pouch possibly supports embryonal development and growth. 17 of the 50 predominating OTUs in the whole study were specific for the paternal brood pouch community (NP, EP, MP, LP). Among those bacteria almost all classified OTUs are known as commensal fish gastrointestinal microbiota (*Acinetobacter, Ruegeria, Marinomonas, Phaeobacter, Alteromonas, Microbacterium, Marinobacter, Cupriavidus* & members of the *Flavobacteriaceae*)^39,41,54–56^. *Marinomonas* and *Cupriavidus* may act as initial colonizers of pipefish larvae^41,56,57^. *Phaecobacter* and *Marinobacterium* may compete with predominant infections in syngnathid pouches^55,58^ and thus boost embryonic well-being and development. *Porticoccus, Sneathiella* & *Oleiphilus* are members of marine waters and are associated to phytoplankton or invertebrates^59–61^.

In mammalian viviparity the embryo is supposed to be kept almost sterile in the mother’s womb, in pipefish the environment could play a pronounced role in the microbial colonisation of the developing embryos. Due to excessive larval growth towards the end of pregnancy and the forthcoming birth, the skin fold that closes the brood pouch upon intake of the eggs, gets chapped, which increases the permeability of the brood pouch. The embryos thus get in contact with the bacteria of the natural habitat (surrounding water). This invasion of environmental microbes could prime or pre-adapt the embryos towards the microbial community, they will encounter in the near future. The shift from the constant microbiota during early and mid-pregnancy in the pouch towards a more variable microbiota during late pregnancy could support the hypothesized invasion of environmental microbes. Drivers of the microbial modulation are *Kiloniella, Aquimarina* and *Ulvibacter*. *Kiloniella* have been previously reported to increase in abundance on the skin of fishes in response to stress^40^, while *Aquimarina* not only belong to fish gut microbiota but also have the potential to inhibit the growth of pathogenic bacteria^62^. *Ulvibacter* belong to *Flavobacteria* that are common bacterial colonizers in the gastrointestinal tract of marine fishes^63^.

A shift in the bacterial community during late pregnancy is further substantiated by a common pool of indicator species shared over all developmental stages, with the exception of the late pregnancy stage. Due to high abundance (under the top 20 most abundant OTUs), these bacteria might be important for the early embryonic development. Among them are *Caldalibacillus, Virgibacillus* and *Pseudoidiomarinae, Nesteronkonia, Bacillaceae* and *Phyllobacterium*. *Virgibacillus* has been reported to have antimicrobial activity, which plays an important role in host defence against pathogenic bacteria in the ovaries or in the pouch^64,65^. These microbes potentially shape initial colonization that occurs prior to the mouth opening, i.e. transovarially, or they are relocated with the paternal nutrient transfer in the pregnancy to the embryos.

This study provides first insight into potential maternal and paternal contribution to offspring microbiota. Syngnathids are an enigmatic model system to assess this, as only the evolution of male pregnancy permits a straightforward differentiation between microbial colonization of the ovary from the colonization during pregnancy. As females produce the eggs and males brood the embryos in their brood pouch, our data simultaneously reflect sex-specific microbial contribution. We identified a microbial community on the eggs in the maternal ovaries that was distinct from all other developmental stages. The bacteria driving this difference are associated to fish mucus (*Brevinema*)^66^ and fish gut microbiota (*Brevinema, Saprospiraceae*)^67,68^.

While highly prevalent in the gonads and on the pipefish eggs, none of these bacteria was abundant during late pregnancy. Promising candidates for a transovarial transfer are thus *Sulfitobacter* and members of the *Flavobacteriaceae*, as they were prevalent both in the gonads and in the pouch during late pregnancy but not in the pouches of non-pregnant males or during early pregnancy. This makes an involvement in both a transfer via eggs into the brood pouch and in initial microbial colonization likely. Both *Sulfitobacter* and *Flavobacteriaceae* are important members of fish larval commensal microbiota^41,58^.

The parental immune system is in close interaction with the microbial community in the gonads and in the parental brood pouch. The identified expansion in species richness during late pregnancy was much more pronounced upon paternal bacterial treatment envoking an immunological activation. This suggests that the immune system of the parents may shape the microbial community in the gonads, the time point when transovarial transfer of microbiota can occur, but also during late pregnancy, when initial microbial gut colonization is most likely to take place. Some of the bacterial indicator species for the gonad and late pregnancy microbial communities are also overrepresented in the microbial community of the gonads from females that were previously injected with heat-killed bacteria (interaction of developmental stage and parental bacteria treatment). In addition to the previously discussed indicator species (*Kiloniella, Aquimarina, Ulvibacter*) *Pseudomonas* is highly prevalent during late pregnancy, if the immunological activation is considered. *Pseudomonas* belongs to the dominant commensal microbiota of marine fish species^58^. *Marinomas* was most abundant during late pregnancy on the embryos of fathers that were previously exposed to bacteria treatment.

The previously described bi-parental immune priming in the pipefish *S. typhle*^30–32^, which entails that the immune status of both parents shapes the immunological performance of their offspring, could directly interact with the microbial community in the ovaries and in the brood pouch. As such, immunological activation imposed over parental bacteria treatment may change the bacterial composition to higher abundance of *Kiloniella, Aquimarina, Ulvibacter* & *Marinomonas* bacteria. These are commensal bacteria with the potential to specifically aid in fighting virulent bacterial infections or alternatively, to boost the offspring immune response.

## Conclusion

Our data suggest a co-adaptation between parental investment, the host microbiota and the immune system during pipefish male pregnancy. Immune defences and the microbial community may be simultaneously transferred from mothers and fathers to the offspring, which can have substantial sex-specific developmental implications that need to be assessed in more detail in the future. Such co-adaptation between microbiota and the vertebrate adaptive immune system are likely to not only enable the clearance of potentially virulent pathogens but also shape the persistence of specific mutualistic microbial communities^33^.

## Material and Methods

### Experimental setup and sampling

Pipefish (females and pregnant/non-pregnant males) were collected from Kiel Fjord (54°44′N; 9°53′E, July 2014) and hosted under Baltic summer conditions (15 PSU; 18 °C) in 500 L tanks. Pipefish were separated according to their sex, and males also according to their developmental stage: 1.group: Females; 2.group: Non-Pregnant males (NP); 3.group: Early Pregnant males (EP) (1–2 weeks); 4.group: Mid Pregnant males (MP) (2–4 weeks); 5.group: Late Pregnant males (LP) (4–6 weeks) (see Fig. 1). 20 females, 20 males per pregnancy stage (20 early, 20 mid 20 late pregnancy), and 40 non-pregnant males were used for the study (in total 120 fish). In the following, 10 females and 10 pregnant males/per pregnancy stage (EP/ MP/ LP) as well as 20 non-pregnant males (undeveloped brood pouch) were randomly chosen and injected with 1:1 mixture of two heat-killed bacteria (*Vibrio spp*. Italy2K3 and *Tenacibaculum maritimum*) according to^32^ to boost their immune system and trigger a parental immune transfer. The bacteria treated individuals and the non-treated pipefish were randomly transferred into 200 L aquaria (3 tank replicates per group (injected *vs*. non-injected), 20 pipefish per tank). Pipefish were fed twice a day with frozen mysids. All tanks were connected through the same filtered local Baltic Seawater (semi-flow through circulation). After 10 days of incubation, the previously immune-challenged pipefish received a secondary injection with the same dosage of heat-killed *Vibrio* bacteria. The ten days between the two paretntal bacteria treatements were chosen to permit sufficient time for an impact of the immune system on the microbiota diversity and simultaneously allow for parental transfer of immunity. 20 hours after the second immune challenge, pipefish were killed by an overdose of an anesthetic (MS-222). The gonads of females were dissected and microbial samples were taken with a sterile swab at the surface of the gonadal eggs. The brood pouch of pregnant pipefish males was opened and microbial samples were taken from the surface of the embryos inside the paternal brood pouch with a sterile swab. The brood pouch tissue of non-pregnant males was opened and microbiota samples were taken with sterile swabs.

### DNA extraction, Library preparation and sequencing

Microbial DNA was extracted with the Dneasy 96 Blood & Tissue Kit (Qiagen) following the manufacturers protocol, with ameliorations according to Franke *et al*. (2017)^69^. The dual-index sequencing strategy developed by^70^ was applied. 16S rRNA genes of samples and a negative (sterile swab) control were amplified (Phusion High-Fidelity DNA Polymerase, Thermo Fisher Scientific) with the labelled primer pairs F515: (AATGATACGGCGACACCGATCTACAC <i5> TATGGTAATTGTGTGCCAGCMGCCGCGGTAA); R806: (CAAGCAGAAGACGGCAACGAGAT <i7> AGTCAGTCAGCCGGACTACHVGGGTWTCTAAT) spanning the hypervariable variable region V4 of the 16S rRNA gene^71^. Forward and reverse primer pairs contained adapters, barcodes, pad and linker sequences as described by^70^. Several negative controls without sample were included per plate. The PCR was carried out under the following conditions: 98 °C for 30 s, 30 cycles of 98 °C for 9 s, followed by 55 °C for 15 s and 72 °C for 20 s with a final elongation step at 72 °C for 10 min. PCR products were purified with the MinElute 96 UF PCR purification kit (Qiagen). DNA concentrations were measured by spectrophotometry (NanoDrop ND-1000, Peqlab). PCR products were pooled to one subpool per plate in an equimolar concentration (~30 ng per sample), respectively, run on a 2% agarose gel, and amplicons of the expected size (~300 bp) extracted using the NucleoSpin gel and PCR clean-up kit (Macherey-Nagel). The extraction products of subpools were fluorometrically quantified (Qubit fluorometer, Invitrogen) and pooled in equimolar concentrations. The amplicon library was sequenced on the Illumina® MiSeq using the MiSeq Reagent Kit v2 500 cycles sequencing chemistry and mixed with Illumina generated phiX control libraries.

### Sequence processing and data analysis

Raw sequences were de-multiplexed using Casava v.1.8.2, assembled and filtered with the software MOTHUR v.1.16.1 according to the MiSeq SOP pipeline^72,73^. Adaptor, tag, and primer sequences were removed from raw sequences. Unique sequence reads were merged and aligned against the SILVA alignment database (release #119)^74,75^. All misaligned sequences not covering the variable region 4 were removed (SILVA alignment position 1968 to 11550)^74,75^. To reduce sequencing noise, a pre-clustering step (2 bp difference) was performed^72^ and chimeric sequences were eliminated by the UCHIME algorithm implemented in MOTHUR^76^. The taxonomy of all sequences was estimated using the *classify.seqs* function in MOTHUR against the Ribosomal Database Project (RDP) training set v.9^77^ with a 80% bootstrap threshold. Non-target sequences (e.g. chloroplasts, mitochondria, eukaryotic 18S rRNA) were removed. The sequences were clustered at the 0.03 difference level to obtain operational taxonomic units (OTUs) with average neighbour algorithm. By randomly taking the same number of sequences from each sample we rarefied the samples to 6000 reads each, which enables to perform and compare diversity measurements at the same sequencing depth (Supplemental Table 1). As the remaining 77 samples were unequally distributed among the treatment groups (Female Control_GO: 6; Female_Injected_Gonads: 10; Male_Control_NP: 12; Male_Injected_NP: 13; Male_Control_Early: 5; Male_Injected_Early: 4; Male_Control_Mid: 8; Male_Injected_Mid: 7; Male_Control_Late: 5; Male_Injected_Late: 7; Supplemental Table S1), specific statistical tests (type III sum of squares, permutational tests) were applied for the analysis. Species richness (number of observed OTUs), estimated species richness (Chao 1 Index, corrected for sample size) and species diversity (Shannon Index and inverse Simpson) were calculated with the *summary.single* command implemented in MOTHUR based on a dataset subsampled to a number of 6000 reads per sample (Supplemental Table S1). For visualization and interpretation of the microbial community data, we used standardized 97%-OTUs for relative abundance (Bray-Curtis distance matrix) analyses.

### Statistical Analysis

All statistical tests and visualizations were performed using a rarefied subset of 6000 reads/sample in the R environment (R Core Team 2015). The α-diversity measurements species richness (number of observed OTUs), estimated species richness (Chao Index), and species diversity (Shannon Index and inverse Simpson) were analysed fitting for each index a *linear mixed effect model*^34^. For the statistical model we applied the fixed interaction term ‘developmental stage’ x ‘bacteria treatment’, ‘tank’ was included as random term. LMER models were performed with the *lmer f*unction implemented in the *lme4* package of R^78^ by using type III sum of squares and Satterthwaite approximation for the degrees of freedom. For multiple comparisons of fixed and interaction terms all significant LMERs were followed by post-hoc t-tests with the *ghlt* function associated in the *multcomp* package of R^79^.

β-diversity measurements were assessed to analyse bacterial communities between tissues and treatment groups based on abundance (Bray-Curtis) of shared 97%-OTUs with the *vegan* package v. 2.3-0 in R^80^. We evaluated changes of microbiota at the surface of the eggs in the ovaries, and at surfaces of increasing developmental embryonal stages in combination with parental bacteria treatment by applying a permutational multivariate analyses of variance (perMANOVA) with the *adonis* function of the *vegan* package in R^81^. PERMANOVA models were based on abundance (Bray-Curtis) data of all OTUs identified in the samples, applying ‘developmental stage’ × ‘bacteria treatment’ as fixed factors and stratifying 10000 permutations within each tank replicate. Visualization of variations in microbiota among developmental stages (gonads, brood pouch of non-pregnant, early, mid and late pregnant males) and parental bacteria treatments were assessed with analysis of principal coordinates (PCoA) based on bray Curtis distance matrix using the *pcoa* function from the *ape* R-package^82–84^. A PCoA is based on eigenvalue equation but can use any dissimilarity index and distances between points in the plot reflecting original distances^85^. Hypothesis-based treatments were added as dispersion ellipses to the ordination plots with 0.95 confidence intervals. We extracted the principle coordinates (scores) of the first two axes and fitted a linear mixed-effect model for each single axis by applying the *lmer* function implemented in the *lme4* package of R^86^. The scores of the first two principle components were used for the statistical analysis to attain the projection that accounts for the most relevant variation^87^. We fitted a linear mixed-effect model using ‘developmental stage’ and ‘bacteria treatment’ as fixed factors and ‘tank’ as random term. The linear mixed-effect model was performed with type III sum of squares and Satterthwaite approximation for degrees of freedom^86^. Significant axes were followed by *Tukey HSD* post-hoc test with the *ghlt* function associated in the *multcomp* package of R^79^ to attain pairewise comparisons between specific treatment groups.

Bacterial distribution patterns and diversity were based on the 50 most abundant OTUs that contribute 90% of all sequence counts (Indicator species on all OTUs can be found in Supplemental Table S3). Indicator bacteria species and associations between species (OTUs) and tissue of interest (gonads, brood pouch of non-pregnant, early, mid and late pregnant males in combination with parental bacteria treatment *vs.* no bacteria treatment), were identified with the package *indicspecies* in R based on 10000 permutations^38^. By drawing a *biplot* (factor map) with the *factoextra* package implemented in R^88^ in which a colour gradient highlights most important species (OTUs), we visualized the contribution of variance (%). In addition, similarity-clustered heatmaps of the 50 most abundant OTU species were conducted. Heatmaps were illustrated with the *aheatmap* function of the NMF package in R by applying an “euclidean” distance and complete linkage method for hierarchical clustering.

### Ethics approval and consent to participate

All animals were handled according to the animal welfare laws of Germany, under a permit of the “Ministerium für Landwirtschaft, Umwelt und ländliche Räume des Landes Schleswig Holstein” called “Komparative Vergleichsstudie von Immunantworts-Transfer von Eltern zu Nachkommen in Fischarten mit extremer Brutpflege”.

## Supplementary information

Supplement

## Data Availability

All data files of the manuscript have been deposited on PANGAEA Server with the Accession Number 10.1594/PANGAEA.896080.
