# Supplementary material for: Microbial embryonal colonization during pipefish male pregnancy
Source: Sci Rep. 2019 Jan 9;9:3. doi: 10.1038/s41598-018-37026-3 (PMC6327025; doi:10.1038/s41598-018-37026-3)
Supplement: Supplementary file 1 — Supplement [file 41598_2018_37026_MOESM1_ESM.docx]

**Microbial embryonal colonization during pipefish male pregnancy**

**Anne Beemelmanns^1^, Maude Poirier^1^, Till Bayer^1^, Sven Kuenzel^2^ & Olivia Roth^1, *^**

^1^GEOMAR Helmholtz-Centre for Ocean Research Kiel, Evolutionary Ecology of Marine Fishes, Düsternbrooker Weg 20, 24105 Kiel, Germany

^2^Max-Planck Institute for Evolutionary Biology, August-Thienemann-Str. 2, 24306 Plön, Germany

**Supplemental Table S1:** **Sample sequence statistics and α-diversity**. Sequence and OUT of 97%-OTUs (subsampling size based on the sample with the fewest sequences emphasised in bold with 61906reads). In the following are listed coverage, species richness (average OTUs), species diversity estimates (Shannon and Inverse simpson), estimated species richness (Chao) each are average values with standard error. Alpha diversity estimates are based on subsampled dataset (6000). Analysis conducted in mother with collect.single command.

| **Sample ID** | **Tank** | **Sex** | **Bac** | **Stage** | **Pregnancy** | **#Sequences** | **Coverage** | **Average OTUs** | **Npshannon** | **Invsimpson** | **Chao** |
| --- | --- | --- | --- | --- | --- | --- | --- | --- | --- | --- | --- |
| **Anne_Mixed_D11_FC_Gonads** | TC1 | F | C | Gonads | Gonads | 25463 | 0.997 | 56.56 | 3.92±0.02 | 20.33±0.7 | 185.4±8.45 |
| **Anne_Mixed_D12_FC_Gonads** | TC2 | F | C | Gonads | Gonads | 18783 | 0.996 | 81.25 | 3.17±0.03 | 11.89±0.3 | 106.87±8.4 |
| **Anne_Mixed_E1_FC_Gonads** | TC3 | F | C | Gonads | Gonads | 57216 | 0.992 | 170.93 | 3.69±0.03 | 14.41±0.56 | 180.07±11.54 |
| **Anne_Mixed_E2_FC_Gonads** | TC3 | F | C | Gonads | Gonads | 12445 | 0.995 | 96.02 | 3.87±0.03 | 22.12±0.76 | 197.42±12.61 |
| **Anne_Mixed_E6_FC_Gonads** | TC1 | F | C | Gonads | Gonads | 17238 | 0.997 | 95.62 | 3.84±0.03 | 17.27±0.62 | 190.7±11.08 |
| **Anne_Mixed_E8_FC_Gonads** | TC2 | F | C | Gonads | Gonads | 17888 | 0.997 | 51.09 | 2.82±0.03 | 8.08±0.21 | 98.14±9.25 |
| **Anne_Mixed_E10_FI_Gonads** | TI1 | F | I | Gonads | Gonads | 14022 | 0.997 | 85.82 | 2.89±0.03 | 7.56±0.22 | 100.95±5.23 |
| **Anne_Mixed_E11_FI_Gonads** | TI3 | F | I | Gonads | Gonads | 18945 | 0.998 | 58.13 | 2.61±0.02 | 7.19±0.1 | 93.66±14.5 |
| **Anne_Mixed_E12_FI_Gonads** | TI2 | F | I | Gonads | Gonads | 22544 | 0.995 | 61.92 | 2.23±0.03 | 4.78±0.11 | 65.94±9.22 |
| **Anne_Mixed_E9_FI_Gonads** | TI1 | F | I | Gonads | Gonads | 43679 | 0.99 | 158.23 | 2.04±0.03 | 4.6±0.08 | 59.42±7.97 |
| **Anne_Mixed_F1_FI_Gonads** | TI3 | F | I | Gonads | Gonads | 21928 | 0.996 | 114.69 | 2.71±0.03 | 7.49±0.24 | 85.11±8.89 |
| **Anne_Mixed_F2_FI_Gonads** | TI2 | F | I | Gonads | Gonads | 49196 | 0.987 | 168.56 | 2.72±0.02 | 7.66±0.12 | 89.65±12.35 |
| **Anne_Mixed_F3_FI_Gonads** | TI3 | F | I | Gonads | Gonads | 23042 | 0.996 | 90.19 | 2.94±0.03 | 8.07±0.26 | 104.06±8.44 |
| **Anne_Mixed_F4_FI_Gonads** | TI1 | F | I | Gonads | Gonads | 29253 | 0.995 | 93.06 | 2.1±0.02 | 4.55±0.09 | 74.3±23.45 |
| **Anne_Mixed_F5_FI_Gonads** | TI2 | F | I | Gonads | Gonads | 6549 | 0.996 | 46.88 | 2.55±0.02 | 6.86±0.16 | 59.61±6.53 |
| **Anne_Mixed_F6_FI_Gonads** | TI1 | F | I | Gonads | Gonads | 22421 | 0.99 | 170.71 | 2.79±0.02 | 9.19±0.24 | 66.11±8.59 |
| **Anne_Pl1_A1_MC_NP** | TC1 | M | C | NP | Non-pregnant | 9449 | 0.994 | 70.9 | 2.33±0.03 | 5.33±0.14 | 65.28±6.72 |
| **Anne_Pl1_A2_MC_NP** | TC2 | M | C | NP | Non-pregnant | 13715 | 0.997 | 57.43 | 3.03±0.02 | 10.96±0.33 | 83.88±7.84 |
| **Anne_Pl1_A3_MC_NP** | TC2 | M | C | NP | Non-pregnant | 11741 | 0.996 | 56.05 | 2.83±0.02 | 8.55±0.25 | 69.67±6.66 |
| **Anne_Pl1_A4_MC_NP** | TC3 | M | C | NP | Non-pregnant | 6278 | 0.995 | 99.96 | 3.05±0.02 | 10.44±0.22 | 104.41±20.26 |
| **Anne_Pl1_A5_MC_NP** | TC2 | M | C | NP | Non-pregnant | 15556 | 0.996 | 102.96 | 2.58±0.02 | 5.46±0.15 | 85.96±7.45 |
| **Anne_Pl1_A6_MC_NP** | TC3 | M | C | NP | Non-pregnant | 15950 | 0.995 | 78.42 | 2.68±0.03 | 7.75±0.22 | 79.86±11.62 |
| **Anne_Pl1_A7_MC_NP** | TC3 | M | C | NP | Non-pregnant | 17792 | 0.994 | 67.63 | 2.11±0.02 | 4.59±0.06 | 84.15±12.37 |
| **Anne_Pl1_B2_MC_NP** | TC1 | M | C | NP | Non-pregnant | 18741 | 0.995 | 72.64 | 2.59±0.02 | 7.27±0.18 | 63.62±6.14 |
| **Anne_Pl1_B4_MC_NP** | TC2 | M | C | NP | Non-pregnant | 15657 | 0.994 | 89.49 | 2.63±0.02 | 7.28±0.18 | 74.87±7.61 |
| **Anne_Pl1_B5_MC_NP** | TC3 | M | C | NP | Non-pregnant | 16748 | 0.995 | 79.72 | 2.39±0.03 | 5.62±0.13 | 71.89±6.04 |
| **Anne_Pl1_B6_MC_NP** | TC3 | M | C | NP | Non-pregnant | 11092 | 0.997 | 57.56 | 3.36±0.02 | 14.83±0.34 | 111.27±8.48 |
| **Anne_Pl1_B8_MC_NP** | TC1 | M | C | NP | Non-pregnant | **6190** | 0.996 | 72.36 | 3.24±0.03 | 11.39±0.37 | 109.98±6.2 |
| **Anne_Pl1_B10_Mi_NP** | TI1 | M | I | NP | Non-pregnant | 9130 | 0.998 | 54.22 | 2.12±0.03 | 3.25±0.08 | 87.26±12.81 |
| **Anne_Pl1_C1_Mi_NP** | TI1 | M | I | NP | Non-pregnant | 17906 | 0.993 | 78.99 | 2.55±0.02 | 7.29±0.18 | 83.87±10.94 |
| **Anne_Pl1_C12_Mi_NP** | TI3 | M | I | NP | Non-pregnant | 27840 | 0.995 | 40.16 | 2.99±0.02 | 11.19±0.3 | 91.06±9.52 |
| **Anne_Pl1_C2_Mi_NP** | TI2 | M | I | NP | Non-pregnant | 32211 | 0.994 | 73.51 | 2.57±0.02 | 6.61±0.12 | 67.16±5.51 |
| **Anne_Pl1_C4_Mi_NP** | TI1 | M | I | NP | Non-pregnant | 15780 | 0.995 | 64.64 | 2.98±0.03 | 8.07±0.28 | 103.09±9.14 |
| **Anne_Pl1_C5_Mi_NP** | TI3 | M | I | NP | Non-pregnant | 16788 | 0.996 | 68.56 | 2.74±0.03 | 7.53±0.19 | 89.48±7.91 |
| **Anne_Pl1_C6_Mi_NP** | TI1 | M | I | NP | Non-pregnant | 15251 | 0.995 | 65.7 | 1.7±0.02 | 3.29±0.04 | 77.08±16.73 |
| **Anne_Pl1_C7_Mi_NP** | TI2 | M | I | NP | Non-pregnant | 20262 | 0.994 | 82.64 | 2.18±0.02 | 4.77±0.1 | 61.34±8.13 |
| **Anne_Pl1_C8_Mi_NP** | TI1 | M | I | NP | Non-pregnant | 20585 | 0.997 | 58.17 | 2.48±0.02 | 5.67±0.15 | 62.54±8.52 |
| **Anne_Pl1_D1_MI_NP** | TI3 | M | I | NP | Non-pregnant | 12427 | 0.996 | 74.94 | 2.5±0.02 | 8.18±0.14 | 60.32±8.14 |
| **Anne_Pl1_D2_Mi_NP** | TI2 | M | I | NP | Non-pregnant | 12222 | 0.998 | 64.14 | 3.02±0.02 | 9.68±0.22 | 107.12±13.74 |
| **Anne_Pl1_D3_Mi_NP** | TI3 | M | I | NP | Non-pregnant | 8899 | 0.996 | 77.11 | 3.03±0.02 | 11.48±0.29 | 100.31±11.42 |
| **Anne_Pl1_D4_Mi_NP** | TI2 | M | I | NP | Non-pregnant | 18148 | 0.996 | 65.98 | 3.66±0.02 | 17.26±0.38 | 166.91±7.54 |
| **Anne_Pl1_D5_MC_Early** | TC1 | M | C | Early | Pregnant | 8632 | 0.996 | 51.87 | 3.48±0.02 | 15.96±0.5 | 131.73±12.59 |
| **Anne_Pl1_D6_MC_Early** | TC2 | M | C | Early | Pregnant | 40926 | 0.994 | 61.35 | 3.46±0.02 | 19.06±0.43 | 107.72±9.12 |
| **Anne_Pl1_D7_MC_Early** | TC3 | M | C | Early | Pregnant | 11049 | 0.998 | 55.46 | 1.02±0.03 | 1.56±0.03 | 57.65±13.67 |
| **Anne_Pl1_D8_MC_Early** | TC3 | M | C | Early | Pregnant | 10390 | 0.997 | 52.22 | 2.71±0.03 | 7.5±0.21 | 99.85±13.41 |
| **Anne_Pl1_E2_MC_Early** | TC2 | M | C | Early | Pregnant | 9252 | 0.997 | 53.1 | 2.63±0.03 | 6.08±0.18 | 87.03±10.7 |
| **Anne_Pl1_E4_MI_Early** | TI3 | M | I | Early | Pregnant | 13272 | 0.996 | 66.36 | 3.31±0.03 | 14.17±0.44 | 120.08±11.98 |
| **Anne_Pl1_E5_MI_Early** | TI3 | M | I | Early | Pregnant | 15662 | 0.996 | 64.97 | 2.18±0.03 | 4.08±0.1 | 73.23±8.05 |
| **Anne_Pl1_E6_MI_Early** | TI1 | M | I | Early | Pregnant | 10400 | 0.997 | 62.48 | 2.61±0.02 | 7.85±0.19 | 69.42±8.72 |
| **Anne_Pl1_E7_MI_Early** | TI2 | M | I | Early | Pregnant | 16827 | 0.994 | 83.62 | 2.76±0.02 | 9.73±0.19 | 84.19±9.29 |
| **Anne_Pl1_F1_MC_Middle** | TC2 | M | C | Middle | Pregnant | 6753 | 0.992 | 98.94 | 1.66±0.03 | 2.25±0.04 | 94.54±14.39 |
| **Anne_Pl1_F10_MC_Middle** | TC1 | M | C | Middle | Pregnant | 19291 | 0.997 | 54.5 | 1.25±0.01 | 1.8±0.01 | 125.61±8.08 |
| **Anne_Pl1_F2_MC_Middle** | TC1 | M | C | Middle | Pregnant | 11253 | 0.996 | 63.28 | 2.09±0.02 | 3.78±0.08 | 91.39±15.53 |
| **Anne_Pl1_F3_MC_Middle** | TC3 | M | C | Middle | Pregnant | 9019 | 0.994 | 95 | 2.91±0.01 | 11.07±0.11 | 87.06±8.17 |
| **Anne_Pl1_F4_MC_Middle** | TC2 | M | C | Middle | Pregnant | 20585 | 0.996 | 59.15 | 2.45±0.02 | 6.58±0.13 | 80.7±13.07 |
| **Anne_Pl1_F5_MC_Middle** | TC1 | M | C | Middle | Pregnant | 13883 | 0.995 | 71.65 | 2.84±0.02 | 8.12±0.2 | 77.82±8.51 |
| **Anne_Pl1_F6_MC_Middle** | TC3 | M | C | Middle | Pregnant | 14428 | 0.995 | 82.34 | 1.88±0.02 | 4.02±0.08 | 46.53±8.58 |
| **Anne_Pl1_F7_MC_Middle** | TC1 | M | C | Middle | Pregnant | 20372 | 0.992 | 110.94 | 2.91±0.02 | 11.26±0.26 | 78.41±11.25 |
| **Anne_Pl1_G1_Mi_Middle** | TI2 | M | I | Middle | Pregnant | 11097 | 0.994 | 71.55 | 3.27±0.03 | 10.36±0.33 | 126.97±8.96 |
| **Anne_Pl1_G2_Mi_Middle** | TI1 | M | I | Middle | Pregnant | 10453 | 0.995 | 89.32 | 1.77±0.04 | 2.26±0.06 | 173.5±36.66 |
| **Anne_Pl1_G3_Mi_Middle** | TI2 | M | I | Middle | Pregnant | 10560 | 0.995 | 61.59 | 2.39±0.03 | 5.73±0.12 | 223.92±41.53 |
| **Anne_Pl1_G4_Mi_Middle** | TI3 | M | I | Middle | Pregnant | 7679 | 0.997 | 70.02 | 1.43±0.04 | 1.84±0.04 | 170.06±40.83 |
| **Anne_Pl1_G5_Mi_Middle** | TI2 | M | I | Middle | Pregnant | 12390 | 0.998 | 38.75 | 2.83±0 | 10.28±0.03 | 73.63±2.67 |
| **Anne_Pl1_G6_Mi_Middle** | TI1 | M | I | Middle | Pregnant | 10611 | 0.995 | 63.65 | 0.39±0.03 | 1.1±0.01 | 99.16±37.36 |
| **Anne_Pl1_G7_Mi_Middle** | TI3 | M | I | Middle | Pregnant | 12767 | 0.994 | 114.08 | 2.67±0.03 | 7.04±0.18 | 101.13±11.52 |
| **Anne_No_85_MC_Late** | TC2 | M | C | Late | Pregnant | 7256 | 0.992 | 69.01 | 3.39±0.03 | 11.94±0.42 | 122.61±6.74 |
| **Anne_No_86_MC_Late** | TC3 | M | C | Late | Pregnant | 7103 | 0.993 | 64.79 | 2.13±0.03 | 3.35±0.09 | 91.66±11.91 |
| **Anne_Pl1_H3_MC_Late** | TC2 | M | C | Late | Pregnant | 15785 | 0.996 | 62.73 | 2.25±0.03 | 4.31±0.12 | 78.62±9.72 |
| **Anne_Pl1_H5_MC_Late** | TC3 | M | C | Late | Pregnant | 15572 | 0.995 | 61.81 | 0.61±0.03 | 1.2±0.01 | 92.57±26.43 |
| **Anne_Pl1_H6_MC_Late** | TI2 | M | C | Late | Pregnant | 14270 | 0.997 | 61.98 | 3.05±0.03 | 11.21±0.29 | 105.86±10.67 |
| **Anne_L_97_MI_Late** | TI1 | M | I | Late | Pregnant | 306053 | 0.992 | 44.44 | 2.64±0.03 | 6.32±0.16 | 92.49±6.68 |
| **Anne_L_99_Mi_Late** | TI2 | M | I | Late | Pregnant | 132766 | 0.985 | 94.18 | 2.26±0.03 | 5.13±0.12 | 65.28±8.46 |
| **Anne_D10_Mi_Late** | TI3 | M | I | Late | Pregnant | 81218 | 0.979 | 114.87 | 2.59±0.03 | 5.93±0.15 | 93.53±11.59 |
| **Anne_D8_Mi_Late** | TI1 | M | I | Late | Pregnant | 98658 | 0.985 | 85.62 | 2.92±0.02 | 9.99±0.21 | 87.03±10.41 |
| **Anne_F7_Mi_Late** | TI2 | M | I | Late | Pregnant | 96436 | 0.992 | 49.84 | 2.06±0.02 | 4.78±0.08 | 45.24±6.83 |
| **Anne_No_91_Mi_Late** | TI3 | M | I | Late | Pregnant | 10966 | 0.993 | 82.87 | 2.64±0.02 | 7.91±0.18 | 82.12±12.87 |
| **Anne_Pl1_H7_Mi_Late** | TI2 | M | I | Late | Pregnant | 24466 | 0.994 | 89.67 | 2.22±0.03 | 4.72±0.11 | 72.39±8.28 |

**Supplemental Table S2: Most abundant 50 OTUs** with taxonomic description.

|  | **OTU** | **[%]** | **Phylum2** | **Phylum3** | **Phylum4** | **Phylum5** | **Phylum6** |
| --- | --- | --- | --- | --- | --- | --- | --- |
| 1 | Otu00001 | 28.1 | Proteobacteria | Gammaproteobacteria | Oceanospirillales | Oceanospirillaceae | Marinomonas |
| 2 | Otu00002 | 10.3 | Proteobacteria | Gammaproteobacteria | Oceanospirillales | Halomonadaceae | Halomonas |
| 3 | Otu00003 | 6.2 | Firmicutes | Bacilli | Bacillales | Bacillaceae_1 | Aeribacillus |
| 4 | Otu00004 | 5.8 | Proteobacteria | Alphaproteobacteria | Rhodobacterales | Rhodobacteraceae | Ruegeria |
| 5 | Otu00005 | 4.5 | Bacteroidetes | Bacteroidetes | Bacteroidetes_unclassified | Bacteroidetes_unclassified | unclassified |
| 6 | Otu00006 | 3.8 | Actinobacteria | Actinobacteria | Actinomycetales | Micrococcaceae | Nesterenkonia |
| 7 | Otu00007 | 2.6 | Proteobacteria | Alphaproteobacteria | Rhodospirillales | Rhodospirillaceae | unclassified |
| 8 | Otu00008 | 2.3 | Proteobacteria | Gammaproteobacteria | Oceanospirillales | Oceanospirillaceae | Marinomonas |
| 9 | Otu00009 | 2.1 | Firmicutes | Bacilli | Bacillales | Bacillales_incertae_sedis | Caldalkalibacillus |
| 10 | Otu00010 | 2.1 | Proteobacteria | Gammaproteobacteria | Alteromonadales | Pseudoalteromonadaceae | Pseudoalteromonas |
| 11 | Otu00011 | 1.7 | Proteobacteria | Proteobacteria | Proteobacteria_unclassified | Proteobacteria_unclassified | unclassified |
| 12 | Otu00012 | 1.6 | Proteobacteria | Gammaproteobacteria | Vibrionales | Vibrionaceae | Vibrio |
| 13 | Otu00013 | 1.4 | Proteobacteria | Alphaproteobacteria | Rhodobacterales | Rhodobacteraceae | Phaeobacter |
| 14 | Otu00014 | 1.3 | Spirochaetes | Spirochaetes | Spirochaetales | Brevinemataceae | Brevinema |
| 15 | Otu00015 | 1.2 | Proteobacteria | Alphaproteobacteria | Rhodobacterales | Rhodobacteraceae | unclassified |
| 16 | Otu00016 | 1.1 | Bacteroidetes | Flavobacteria | Flavobacteriales | Flavobacteriaceae | Aquimarina |
| 17 | Otu00017 | 1.0 | Actinobacteria | Actinobacteria | Actinomycetales | Microbacteriaceae | Microbacterium |
| 18 | Otu00018 | 0.8 | Proteobacteria | Alphaproteobacteria | Kiloniellales | Kiloniellaceae | Kiloniella |
| 19 | Otu00019 | 0.7 | Proteobacteria | Alphaproteobacteria | Rhodobacterales | Rhodobacteraceae | Litoreibacter |
| 20 | Otu00020 | 0.7 | Bacteroidetes | Sphingobacteria | Sphingobacteriales | Saprospiraceae | unclassified |
| 21 | Otu00021 | 0.7 | Firmicutes | Bacilli | Bacillales | Bacillaceae_1 | unclassified |
| 22 | Otu00022 | 0.7 | Firmicutes | Bacilli | Bacillales | Bacillaceae_2 | Virgibacillus |
| 23 | Otu00023 | 0.6 | Bacteroidetes | Flavobacteria | Flavobacteriales | Flavobacteriaceae | unclassified |
| 24 | Otu00024 | 0.6 | Bacteroidetes | Flavobacteria | Flavobacteriales | Flavobacteriaceae | Ulvibacter |
| 25 | Otu00025 | 0.6 | Firmicutes | Bacilli | Bacillales | Bacillales_incertae_sedis | Caldalkalibacillus |
| 26 | Otu00026 | 0.6 | Proteobacteria | Alphaproteobacteria | Rhodobacterales | Rhodobacteraceae | Sulfitobacter |
| 27 | Otu00027 | 0.5 | Proteobacteria | Alphaproteobacteria | Sneathiellales | Sneathiellaceae | Sneathiella |
| 28 | Otu00028 | 0.5 | Bacteroidetes | Flavobacteria | Flavobacteriales | Flavobacteriaceae | unclassified |
| 29 | Otu00029 | 0.5 | Proteobacteria | Gammaproteobacteria | Alteromonadales | Alteromonadaceae | Alteromonas |
| 30 | Otu00030 | 0.5 | Proteobacteria | Gammaproteobacteria | Gammaproteobacteria_ | Gammaproteobacteria_ | Porticoccus |
| 31 | Otu00031 | 0.5 | Spirochaetes | Spirochaetes | Spirochaetales | Brevinemataceae | Brevinema |
| 32 | Otu00032 | 0.4 | Proteobacteria | Betaproteobacteria | Burkholderiales | Burkholderiaceae | Cupriavidus |
| 33 | Otu00033 | 0.4 | Bacteroidetes | Flavobacteria | Flavobacteriales | Flavobacteriaceae | unclassified |
| 34 | Otu00034 | 0.4 | Proteobacteria | Gammaproteobacteria | Pseudomonadales | Pseudomonadaceae | Pseudomonas |
| 35 | Otu00035 | 0.4 | Proteobacteria | Gammaproteobacteria | Pseudomonadales | Moraxellaceae | Acinetobacter |
| 36 | Otu00036 | 0.3 | Firmicutes | Bacilli | Bacillales | Staphylococcaceae | Staphylococcus |
| 37 | Otu00037 | 0.3 | Proteobacteria | Gammaproteobacteria | Alteromonadales | Idiomarinaceae | Pseudidiomarina |
| 38 | Otu00038 | 0.3 | Bacteroidetes | Sphingobacteria | Sphingobacteriales | Saprospiraceae | Lewinella |
| 39 | Otu00039 | 0.2 | Proteobacteria | Alphaproteobacteria | Rhodobacterales | Rhodobacteraceae | Ahrensia |
| 40 | Otu00040 | 0.2 | Proteobacteria | Alphaproteobacteria | Rhizobiales | Phyllobacteriaceae | Phyllobacterium |
| 41 | Otu00041 | 0.2 | Proteobacteria | Gammaproteobacteria | Alteromonadales | Idiomarinaceae | Idiomarina |
| 42 | Otu00042 | 0.2 | Proteobacteria | Gammaproteobacteria | Pseudomonadales | Pseudomonadaceae | Pseudomonas |
| 43 | Otu00043 | 0.2 | Proteobacteria | Gammaproteobacteria | Gammaprotbac_unclass. | Gammaprotbac_unclass. | unclassified |
| 44 | Otu00044 | 0.2 | Spirochaetes | Spirochaetes | Spirochaetales | Brevinemataceae | Brevinema |
| 45 | Otu00045 | 0.2 | Proteobacteria | Gammaproteobacteria | Oceanospirillales | Halomonadaceae | Cobetia |
| 46 | Otu00046 | 0.2 | Proteobacteria | Gammaproteobacteria | Gammaproteobacteria | Gammaproteobacteria_family_ | Porticoccus |
| 47 | Otu00047 | 0.2 | Firmicutes | Bacilli | Lactobacillales | Streptococcaceae | Streptococcus |
| 48 | Otu00048 | 0.2 | Proteobacteria | Gammaproteobacteria | Gammaprotbac_unclass. | Gammaprotbac_unclass. | unclassified |
| 49 | Otu00049 | 0.2 | Proteobacteria | Gammaproteobacteria | Oceanospirillales | Oleiphilaceae(96) | Oleiphilus |
| 50 | Otu00050 | 0.2 | Proteobacteria | Gammaproteobacteria | Alteromonadales | Alteromonadaceae | Marinobacterium |
|  | TOTAL | 90% |  |  |  |  |  |

**Supplemental Table S3:** **List of species associated to each combination (Indicator Species) to identify associations between species (OTUs) and combination of sites (tissue of brood-pouch with increasing pregnancy)**. A indicator species analysis was performed with the package ìndicspecies` implemented in R based on 1000 permutations (Dufrene & Legendre 1997,Caceres & Legendre 2009) on all OTUS (1416). Listed are the OTUs, the OTU Taxonomy, the indicator value (IndVal) index with is the product of A (specificity) and B (sensitivity) probabilities and significance values (pvalues). Only OTUS with an IndVal value higher than >0.5 (A & B) were considered to exclude bacterial taxa which were only present in one or few tissue samples. OTUs colored in red are among the most abundant 50 OTUs (Table 4).

| **# OTU** | **Taxonomy OTUs** | | | | | Stats | p-value | sign |
| --- | --- | --- | --- | --- | --- | --- | --- | --- |
| **Indicator species associated to Gonads** | | | | | | | | |
| **Otu00020** | Bacteroidetes | Sphingobacteria | Sphingobacteriales | Saprospiraceae | **Saprospiraceae** | 0.96 | 0.001 | *** |
| **Otu00104** | Proteobacteria | Alphaproteobacteria | Rhizobiales | Hyphomicrobiaceae | **Hyphomicrobium** | 0.87 | 0.001 | *** |
| **Otu00091** | Bacteroidetes | Flavobacteria | Flavobacteriales | Flavobacteriaceae | **Flavobacteriaceae** | 0.83 | 0.001 | *** |
| **Otu00059** | Proteobacteria | Alphaproteobacteria | Rhodobacterales | Rhodobacteraceae | **Rhodobacteraceae(92)** | 0.83 | 0.001 | *** |
| **Otu00106** | Bacteroidetes | Flavobacteria | Flavobacteriales | Cryomorphaceae | **Lishizhenia(98)** | 0.81 | 0.001 | *** |
| **Otu00068** | Proteobacteria | Alphaproteobacteria | Rhizobiales | Phyllobacteriaceae | **Hoeflea(61)** | 0.79 | 0.001 | *** |
| **Otu00108** | Bacteroidetes | Flavobacteria | Flavobacteriales | Flavobacteriales(84) | **Flavobacteriales(84)** | 0.79 | 0.001 | *** |
| **Otu00097** | Proteobacteria | Alphaproteobacteria | Rhodobacterales(98) | Rhodobacteraceae(98) | **Rhodobacteraceae(95)** | 0.76 | 0.001 | *** |
| **Otu00038** | Bacteroidetes | Sphingobacteria | Sphingobacteriales | Saprospiraceae | **Lewinella** | 0.75 | 0.001 | *** |
| **Otu00093** | Proteobacteria | Gammaproteobacteria | Gammaproteobacteria(96) | Gammaproteobacteria(96) | **Gammaproteobacteria(96)** | 0.75 | 0.001 | *** |
| **Otu00126** | Bacteroidetes | Flavobacteria | Flavobacteriales | Cryomorphaceae | **Crocinitomix** | 0.73 | 0.001 | *** |
| **Otu00094** | Proteobacteria | Alphaproteobacteria | Sphingomonadales | Sphingomonadaceae | **Sphingopyxis** | 0.72 | 0.001 | *** |
| **Otu00064** | Proteobacteria | Alphaproteobacteria | Rhizobiales(89) | Rhizobiales(89) | **Rhizobiales(89)** | 0.70 | 0.01 | ** |
| **Otu00175** | Proteobacteria | Alphaproteobacteria | Rhodobacterales | Rhodobacteraceae | **Rhodobacteraceae** | 0.70 | 0.001 | *** |
| **Otu00223** | Proteobacteria | Alphaproteobacteria | Rhodobacterales | Rhodobacteraceae | **Rhodobacteraceae** | 0.70 | 0.001 | *** |
| **Otu00072** | Proteobacteria | Gammaproteobacteria | Oceanospirillales | Oceanospirillaceae | **Oceanospirillaceae** | 0.69 | 0.004 | ** |
| **Otu00138** | Bacteroidetes | Sphingobacteria | Sphingobacteriales | Flammeovirgaceae | **Reichenbachiella** | 0.66 | 0.001 | *** |
| **Otu00179** | Proteobacteria | Alphaproteobacteria | Alphaproteobacteria(96) | Alphaproteobacteria(96) | **Alphaproteobacteria(96)** | 0.66 | 0.001 | *** |
| **Otu00125** | Proteobacteria | Alphaproteobacteria | Rhodobacterales | Rhodobacteraceae | **Thalassobacter(99)** | 0.66 | 0.001 | *** |
| **Otu00147** | Proteobacteria | Gammaproteobacteria | Alteromonadales | Alteromonadaceae | **Haliea** | 0.64 | 0.001 | *** |
| **Otu00130** | Proteobacteria | Betaproteobacteria | Betaproteobacteria | Betaproteobacteria | **Betaproteobacteria** | 0.63 | 0.003 | ** |
| **Otu00044** | Spirochaetes | Spirochaetes | Spirochaetales | Brevinemataceae | **Brevinema** | 0.62 | 0.002 | ** |
| **Otu00389** | Proteobacteria | Proteobacteria | Proteobacteria | Proteobacteria | **Proteobacteria** | 0.62 | 0.001 | *** |
| **Otu00117** | Proteobacteria | Alphaproteobacteria | Sphingomonadales | Erythrobacteraceae | **Erythrobacter** | 0.62 | 0.001 | *** |
| **Otu00136** | Proteobacteria | Alphaproteobacteria | Parvularculales | Parvularculaceae | **Parvularcula** | 0.61 | 0.001 | *** |
| **Otu00144** | Proteobacteria | Gammaproteobacteria | Gammaproteobacteria | Gammaproteobacteria | **Gammaproteobacteria** | 0.61 | 0.002 | ** |
| **Otu00187** | Bacteroidetes | Sphingobacteria | Sphingobacteriales | Saprospiraceae | **Haliscomenobacter** | 0.61 | 0.002 | ** |
| **Otu00135** | Proteobacteria | Gammaproteobacteria | Vibrionales | Vibrionaceae | **Photobacterium** | 0.61 | 0.005 | ** |
| **Otu00178** | Bacteroidetes | Flavobacteria | Flavobacteriales | Flavobacteriaceae | **Ulvibacter(58)** | 0.61 | 0.001 | *** |
| **Otu00151** | Actinobacteria | Actinobacteria | Acidimicrobiales | Acidimicrobiaceae | **Acidimicrobiaceae** | 0.61 | 0.002 | ** |
| **Otu00075** | Actinobacteria | Actinobacteria | Acidimicrobiales | Acidimicrobiales | **Acidimicrobiales** | 0.61 | 0.003 | ** |
| **Otu00127** | Planctomycetes | Planctomycetacia(82) | Planctomycetales(82) | Planctomycetaceae(82) | **Planctomycetaceae(82)** | 0.60 | 0.002 | ** |
| **Otu00153** | Bacteroidetes | Sphingobacteria | Sphingobacteriales | Flammeovirgaceae | **Fabibacter** | 0.60 | 0.002 | ** |
| **Otu00065** | Tenericutes | Mollicutes | Mycoplasmatales | Mycoplasmataceae | **Mycoplasmataceae(99)** | 0.56 | 0.004 | ** |
| **Otu00119** | Proteobacteria | Gammaproteobacteria | Gammaproteobacteria | Gammaproteobacteria | **Thioprofundum(98)** | 0.56 | 0.005 | ** |
| **Otu00193** | Proteobacteria | Alphaproteobacteria | Rhodobacterales | Rhodobacteraceae | **Roseovarius** | 0.56 | 0.002 | ** |
| **Otu00204** | Proteobacteria | Alphaproteobacteria | Rhizobiales | Hyphomicrobiaceae | **Hyphomicrobium** | 0.56 | 0.001 | *** |
| **Otu00233** | Proteobacteria | Alphaproteobacteria | Alphaproteobacteria | Alphaproteobacteria | **Alphaproteobacteria** | 0.56 | 0.003 | ** |
| **Otu00031** | Spirochaetes | Spirochaetes | Spirochaetales | Brevinemataceae | **Brevinema** | 0.56 | 0.014 | * |
| **Otu00152** | Bacteroidetes | Sphingobacteria | Sphingobacteriales | Saprospiraceae | **Saprospiraceae(98)** | 0.55 | 0.003 | ** |
| **Otu00229** | Bacteroidetes | Flavobacteria | Flavobacteriales | Cryomorphaceae | **Owenweeksia** | 0.55 | 0.003 | ** |
| **Otu00086** | Proteobacteria | Gammaproteobacteria | Gammaproteobacteria | Gammaproteobacteria | **Gammaproteobacteria** | 0.55 | 0.011 | * |
| **Otu00121** | Proteobacteria | Gammaproteobacteria | Gammaproteobacteria | Gammaproteobacteria | **Gammaproteobacteria** | 0.52 | 0.034 | * |
| **Indicator species associated to embryos of "Non-pregnant" pregnacy stage** | | | | | | | | |
| **Otu00191** | Proteobacteria | Gammaproteobacteria | Alteromonadales | Alteromonadaceae | **Haliea** | 0.529 | 0.009 | ** |
| **Indicator species associated to embryos of "Early" pregnacy stage** | | | | | |  | | |
| **Otu00423** | Actinobacteria | Actinobacteria | Actinomycetales | Nocardiaceae | **Nocardia** | 0.50 | 0.005 | ** |
| **Indicator species associated to embryos of "Middle" pregnacy stage** | | | | | |  | | |
| **Otu00381** | Proteobacteria | Alphaproteobacteria | Rhizobiales | Rhizobiales | **Rhizobiales** | 0.50 | 0.012 | * |
| **Indicator species associated to embryos of "Late" pregnacy stage** | | | | | |  | | |
| **Otu00018** | Proteobacteria | Alphaproteobacteria | Kiloniellales | Kiloniellaceae | **Kiloniella** | 0.70 | 0.005 | ** |
| **Otu00051** | Bacteroidetes | Flavobacteria | Flavobacteriales | Flavobacteriaceae | **Flavobacteriaceae(96)** | 0.63 | 0.002 | ** |
| **Otu00063** | Proteobacteria | Gammaproteobacteria | Alteromonadales | Colwelliaceae | **Thalassomonas** | 0.59 | 0.004 | ** |
| **Otu00071** | Proteobacteria | Alphaproteobacteria | Rhodospirillales | Rhodospirillaceae | **Thalassospira** | 0.58 | 0.01 | ** |
| **Otu00122** | Proteobacteria | Gammaproteobacteria | Alteromonadales | Colwelliaceae | **Thalassomonas** | 0.57 | 0.002 | ** |
| **Otu00174** | Proteobacteria | Alphaproteobacteria | Rhizobiales | Methylocystaceae | **Terasakiella** | 0.56 | 0.001 | *** |
| **Otu00070** | Proteobacteria | Gammaproteobacteria | Oceanospirillales | Oceanospirillaceae | **Neptunomonas** | 0.56 | 0.021 | * |
| **Otu00107** | Bacteroidetes | Flavobacteria | Flavobacteriales | Flavobacteriaceae | **Maribacter** | 0.55 | 0.002 | ** |
| **Otu00110** | Proteobacteria | Gammaproteobacteria | Oceanospirillales | Oceanospirillaceae | **Amphritea(98)** | 0.53 | 0.016 | * |
| **Otu00319** | Proteobacteria | Gammaproteobacteria | Alteromonadales | Shewanellaceae | **Shewanella** | 0.51 | 0.021 | * |
| **Otu00224** | Bacteroidetes | Sphingobacteria | Sphingobacteriales | Flammeovirgaceae | **Flammeovirgaceae** | 0.51 | 0.017 | * |
| **Otu00120** | Proteobacteria | Gammaproteobacteria | Alteromonadales | Pseudoalteromonadaceae | **Algicola** | 0.50 | 0.007 | ** |
| **Otu00166** | Proteobacteria | Gammaproteobacteria | Oceanospirillales | Oceanospirillaceae(51) | **Oceanospirillaceae(51)** | 0.50 | 0.007 | ** |
| **Otu00237** | Proteobacteria | Gammaproteobacteria | Gammaproteobacteria | Gammaproteobacteria | **Gammaproteobacteria** | 0.50 | 0.007 | ** |
| **Otu00016** | Bacteroidetes | Flavobacteria | Flavobacteriales | Flavobacteriaceae | **Aquimarina** | 0.50 | 0.022 | * |
| **Indicator species associated to tissue of Non-pregnant males and embryos in "Early","Middle", "Late" pregnacy stage** | | | | | | | | |
| **Otu00035** | Proteobacteria | Gammaproteobacteria | Pseudomonadales | Moraxellaceae | **Acinetobacter** | 0.96 | 0.001 | *** |
| **Otu00004** | Proteobacteria | Alphaproteobacteria | Rhodobacterales | Rhodobacteraceae | **Ruegeria(99)** | 0.94 | 0.001 | *** |
| **Otu00008** | Proteobacteria | Gammaproteobacteria | Oceanospirillales | Oceanospirillaceae | **Marinomonas** | 0.93 | 0.001 | *** |
| **Otu00030** | Proteobacteria | Gammaproteobacteria | Gammaproteobacteria | Gammaproteobacteria_ | **Porticoccus** | 0.92 | 0.001 | *** |
| **Otu00013** | Proteobacteria | Alphaproteobacteria | Rhodobacterales | Rhodobacteraceae | **Phaeobacter** | 0.91 | 0.001 | *** |
| **Otu00028** | Bacteroidetes | Flavobacteria | Flavobacteriales | Flavobacteriaceae | **Flavobacteriaceae(99)** | 0.91 | 0.001 | *** |
| **Otu00029** | Proteobacteria | Gammaproteobacteria | Alteromonadales | Alteromonadaceae | **Alteromonas(95)** | 0.90 | 0.001 | *** |
| **Otu00017** | Actinobacteria | Actinobacteria | Actinomycetales | Microbacteriaceae | **Microbacterium** | 0.89 | 0.001 | *** |
| **Otu00078** | Proteobacteria | Alphaproteobacteria | Rhizobiales | Phyllobacteriaceae | **Mesorhizobium** | 0.89 | 0.001 | *** |
| **Otu00079** | Proteobacteria | Betaproteobacteria | Burkholderiales | Burkholderiales | **Tepidimonas(94)** | 0.88 | 0.001 | *** |
| **Otu00027** | Proteobacteria | Alphaproteobacteria | Sneathiellales | Sneathiellaceae | **Sneathiella** | 0.88 | 0.001 | *** |
| **Otu00046** | Proteobacteria | Gammaproteobacteria | Gammaproteobacteria | Gammaproteobacteria | **Porticoccus(66)** | 0.87 | 0.001 | *** |
| **Otu00005** | Bacteroidetes | Bacteroidetes | Bacteroidetes | Bacteroidetes | **Bacteroidetes** | 0.87 | 0.001 | *** |
| **Otu00043** | Proteobacteria | Gammaproteobacteria | Gammaproteobacteria | Gammaproteobacteria | **Gammaproteobacteria** | 0.85 | 0.002 | ** |
| **Otu00048** | Proteobacteria | Gammaproteobacteria | Gammaproteobacteria | Gammaproteobacteria | **Gammaproteobacteria** | 0.85 | 0.001 | *** |
| **Otu00049** | Proteobacteria | Gammaproteobacteria | Oceanospirillales | Oleiphilaceae(96) | **Oleiphilus(96)** | 0.84 | 0.001 | *** |
| **Otu00050** | Proteobacteria | Gammaproteobacteria | Alteromonadales | Alteromonadaceae | **Marinobacterium** | 0.83 | 0.001 | *** |
| **Otu00060** | Proteobacteria | Gammaproteobacteria | Alteromonadales | Colwelliaceae | **Colwellia** | 0.83 | 0.002 | ** |
| **Otu00057** | Proteobacteria | Gammaproteobacteria | Alteromonadales | Alteromonadaceae | **Haliea** | 0.82 | 0.005 | ** |
| **Otu00088** | Proteobacteria | Alphaproteobacteria | Rhodobacterales | Rhodobacteraceae | **Rhodobacteraceae** | 0.79 | 0.002 | ** |
| **Otu00032** | Proteobacteria | Betaproteobacteria | Burkholderiales | Burkholderiaceae | **Cupriavidus** | 0.79 | 0.003 | ** |
| **Otu00055** | Proteobacteria | Alphaproteobacteria | Sneathiellales | Sneathiellaceae | **Sneathiella** | 0.69 | 0.013 | * |
| **Otu00011** | Proteobacteria | Proteobacteria | Proteobacteria | Proteobacteria | **Proteobacteria** | 0.65 | 0.015 | * |
| **Otu00159** | Proteobacteria | Proteobacteria | Proteobacteria | Proteobacteria | **Proteobacteria** | 0.59 | 0.03 | * |
